# Autism spectrum disorder and epileptic encephalopathy: common causes, many questions

**DOI:** 10.1186/s11689-017-9202-0

**Published:** 2017-06-23

**Authors:** Siddharth Srivastava, Mustafa Sahin

**Affiliations:** Department of Neurology, Boston Children’s Hospital, 300 Longwood Avenue, Boston, MA 02115 USA

**Keywords:** Autism spectrum disorder, Epileptic encephalopathy, Mendelian disorders

## Abstract

Epileptic encephalopathies represent a particularly severe form of epilepsy, associated with cognitive and behavioral deficits, including impaired social-communication and restricted, repetitive behaviors that are the hallmarks of autism spectrum disorder (ASD). With the advent of next-generation sequencing, the genetic landscape of epileptic encephalopathies is growing and demonstrates overlap with genes separately implicated in ASD. However, many questions remain about this connection, including whether epileptiform activity itself contributes to the development of ASD symptomatology. In this review, we compiled a database of genes associated with both epileptic encephalopathy and ASD, limiting our purview to Mendelian disorders not including inborn errors of metabolism, and we focused on the connection between ASD and epileptic encephalopathy rather than epilepsy broadly. Our review has four goals: to (1) discuss the overlapping presentations of ASD and monogenic epileptic encephalopathies; (2) examine the impact of the epilepsy itself on neurocognitive features, including ASD, in monogenic epileptic encephalopathies; (3) outline many of the genetic causes responsible for both ASD and epileptic encephalopathy; (4) provide an illustrative example of a final common pathway that may be implicated in both ASD and epileptic encephalopathy. We demonstrate that autistic features are a common association with monogenic epileptic encephalopathies. Certain epileptic encephalopathy syndromes, like infantile spasms, are especially linked to the development of ASD. The connection between seizures themselves and neurobehavioral deficits in these monogenic encephalopathies remains open to debate. Finally, advances in genetics have revealed many genes that overlap in ties to both ASD and epileptic encephalopathy and that play a role in diverse central nervous system processes. Increased attention to the autistic features of monogenic epileptic encephalopathies is warranted for both researchers and clinicians alike.

**Electronic supplementary material:**

The online version of this article (doi:10.1186/s11689-017-9202-0) contains supplementary material, which is available to authorized users.

## Background

Though there is a well-known and interesting connection between autism spectrum disorder (ASD) and epilepsy, the association between ASD and epileptic encephalopathy is particularly intriguing. ASD is a disorder of neurodevelopment characterized by impaired social communication (abnormalities in social-emotional reciprocity, nonverbal communication used for social interaction, and social relationships) as well as restricted, repetitive interests and behaviors [1]. A significant percentage of children with ASD have co-morbid epilepsy, ranging from 9–22% in some studies [2, 3], encompassing a wide range of clinical severity. One of the most severe forms is epileptic encephalopathy, which is characterized by intractable seizures as well as frequent ictal or interictal epileptiform activity that contributes to the cognitive and behavioral phenotype including ASD [4]. This definition is based on evidence that epileptiform itself activity can disrupt brain development through multiple mechanisms such as alteration of neurotransmitter systems and neuronal properties [5]. In some cases, the epilepsy does not have an identified cause, while in others, it is due to an underlying structural/metabolic/genetic etiology (i.e., symptomatic).

When the cause of an epileptic encephalopathy is genetic, it raises the question, what is the nature and extent of the contribution of the gene defect, versus the epilepsy itself, towards the neurobehavioral phenotype? In other words, for different genetic epileptic encephalopathies, what is the autism profile, and how does this profile differ from the core deficits seen in individuals with the same genetic defect who have ASD but not epileptic encephalopathy? One approach to understanding the answers to some of these questions is by first examining the set of genetic causes common to both as well as the associated ASD phenotypes.

Indeed, a growing body of evidence suggests that ASD stems from genetic causes [6–8], such as complex inheritance, chromosomal copy number variation, single gene mutations, or epigenetic changes. Autism twin studies reveal significantly higher concordance rates for monozygotic twins (70–90%) compared to corresponding rates for dizygotic twins (0–10%) [9, 10], while family studies indicate that a child has a much higher risk of developing ASD, compared to the population prevalence, if there is a sibling in the family who is already affected [11]. Moreover, siblings and parents of affected probands have a greater likelihood of exhibiting autistic features strikingly similar to, but milder than, the social and communicative deficits seen in children with ASD [12, 13]. A subset of individuals with ASD have copy number variations (CNVs) affecting gene clusters implicated in neurodevelopment, such as 16p11 microdeletion/microduplication [14], 15q11 duplication [15], and many others [16]. Finally, there is evidence arguing that de novo mutations play a much greater role in ASD than previously expected. Statistical analysis of ASD family data suggests that sporadic autism may stem in part from parental germline mutations targeting any of a number of important loci [17]. These genes are responsible for many fundamental central nervous system (CNS) processes, including synaptic transmission and neuronal plasticity [18].

As with ASD, the genetic landscape of epileptic encephalopathy is expanding. Previous efforts have carefully characterized the phenotype of known genetic disorders that lead to epileptic encephalopathy, such as *CDKL5*-, *SCN1A*-, and *STXBP1*-related disorders. With next-generation sequencing approaches focusing on various epileptic encephalopathy syndromes, such as Lennox–Gastaut syndrome (LGS) and infantile spasms (IS), researchers have identified likely pathogenic variants in numerous other genes that are statistically associated with the underlying phenotype [19, 20]. Disruption to any one of these genes implicated in epileptic encephalopathy may have neurodevelopmental consequences that extend beyond epileptogenesis, influencing the realm of cognition and behavior.

In order to learn more about these causes, we compiled a database of genes associated with both epileptic encephalopathy and ASD. We limited our purview to Mendelian disorders not including inborn errors of metabolism, and we focused on the connection between ASD and epileptic encephalopathy rather than epilepsy broadly, which is the subject of several existing reviews [21, 22].

Specifically, starting with a list of genes implicated in epileptic encephalopathy [23], we searched Pubmed and the Simons Foundation Autism Research Initiative (SFARI) gene (https://gene.sfari.org). For each gene of interest, when searching Pubmed, we used the query, “([gene symbol] AND autism) OR ([gene symbol] AND ASD)”; when searching SFARI Gene, we searched for the gene symbol. From the search results, we reviewed each article and extracted clinical information from it if it demonstrated pathogenic or likely pathogenic variant(s) affecting the gene of interest underlying clinical presentations of ASD and epileptic encephalopathy. Simultaneously, we collected information from studies that demonstrated the presence of pathogenic or likely pathogenic variants affecting the gene of interest underlying clinical presentations of ASD (regardless of other features such as epileptic encephalopathy). Regarding genetic variants, we included cases involving intragenic variants as well as those with CNVs that affected only the gene of interest. Regarding the description of ASD, we included studies that used the following terminology: “autistic features,” “autistic-like features,” “autism-like behavior,” “autistic disorder,” “autism,” and “autism spectrum disorder”. If we were unclear how the study authors defined the presence of ASD or autistic features, we surrounded their terminology in quotation marks; otherwise, we specified the basis for the diagnosis. Regarding the description of epileptic encephalopathy, we included studies that explicitly characterized patients having an “epileptic encephalopathy” or that delineated the presence of a known epileptic encephalopathy syndrome, such as LGS or IS. If the patient had “intractable epilepsy,” then we reviewed the EEG description for features consistent with an epileptic encephalopathy.

Using this data, our review will cover four aims. The first aim is to discuss the overlapping presentations of ASD and monogenic epileptic encephalopathies. The second aim is to examine the impact of the epilepsy itself on neurocognitive features, including ASD, in monogenic epileptic encephalopathies. The third aim is to outline many of the genetic causes responsible for both ASD and epileptic encephalopathy. The final aim is to provide an illustrative example of a final common pathway that may be implicated in both ASD and epileptic encephalopathy.

## Overview of monogenic epileptic encephalopathies

Monogenic epileptic encephalopathies encompass several known epilepsy syndromes characterized by generally severe presentations with varying ages of onset and typical electrographic findings. Some of these syndromes present in the newborn period and respond poorly to treatments; examples include early-onset epileptic encephalopathy, early infantile epileptic encephalopathy, early myoclonic encephalopathy, and epilepsy of infancy with migrating focal seizures. Others, such as IS, Dravet syndrome (DS), and epilepsy with myoclonic-astatic seizures, lead to seizures later in infancy [23]. LGS is defined by the presence of multiple seizure types that emerge between 1–8 years of age [24]. Epilepsy-aphasia spectrum, which contains Landau-Kleffner syndrome (LKS) and continuous spike-wave discharges in slow wave sleep syndrome (CSWSS), has the notable feature of developmental regression [25]. A multitude of single gene defects are associated with different phenotypic classifications of epileptic encephalopathy, reflecting the heterogeneity of this group as a whole [23].

## ASD profile across monogenic epileptic encephalopathies

### ASD prevalence

Among the different genetic causes of epileptic encephalopathy, the prevalence of ASD features is not completely known, but it reaches high levels in some specific instances (Additional file 1: Table S1). Out of the disorders where we can calculate prevalence, the lowest prevalence (6%) occurs in *GRIN2A*-related disorders, which disrupt the N-methyl-D-aspartate (NMDA) receptor, critical for learning and memory (http://www.ncbi.nlm.nih.gov/gene/2903), resulting in epilepsy-aphasia spectrum. Out of 36 patients from 16 families with epileptic aphasia spectrum due to *GRIN2A* alterations, two non-related individuals had “autistic features” and CSWSS. One of the two had global developmental delay without regression; the other had a normal initial developmental period followed by global regression with predominant effects on language [26]. In contrast to *GRIN2A*-related disorders, epileptic encephalopathies associated with mutations in *CDKL5* (encoding cyclin-dependent kinase-like 5), *SCN1A* (encoding sodium voltage-gated channel alpha subunit 1), and *SLC6A1* (encoding GABA transporter 1, which enables GABA re-uptake from the synaptic cleft) have a high co-occurrence of ASD features. In one case series of 10 patients with *CDKL5* mutations, all (100%) patients had “autistic features” in addition to epilepsy, though the cohort was enriched for patients with early-onset epileptic encephalopathy and/or a clinical diagnosis of Rett syndrome with negative *MECP2* sequencing [27]. Out of 15 patients with DS secondary to mutations in *SCN1A*, 11 (or 73%) had a diagnosis of ASD based on Diagnostic and Statistical Manual of Mental Disorders-IV (DSM-IV) criteria, mild-severe intellectual disability (ID), and various seizure types [28]. In a cohort of 7 patients with *SLC6A1* variants underlying myoclonic-atonic epilepsy, 5 (71%) had “autistic features,” mild-severe ID (along with regression in 2 individuals), and a full spectrum of seizure types [29]. Other disorders with relatively high rates of ASD features alongside epileptic encephalopathy include *HCN1*-related epilepsy (67% [30]) and *SIK1*-related epilepsy (50% [31]). *HCN1* mutations affect hyperpolarization-activated, cyclic nucleotide-gated (HCN) channels which regulate pacemaker currents in neurons, resulting in an early infantile epileptic encephalopathy with characteristics of DS initially followed later by the emergence of atypical absences, ID, and ASD features [30]. Defects in salt-inducible kinase 1 (*SIK1*), encoding a serine/threonine protein kinase implicated in signal transduction, cause a spectrum of epileptic encephalopathies including early myoclonic encephalopathy, IS, and Ohtahara syndrome (OS) [31].

Of note, these ASD prevalence numbers are based on descriptive case series comprising at least five patients with variants in the gene of interest who have epileptic encephalopathy. As a result, there is insufficient data to calculate ASD prevalence rates for all the genes in Additional file 1: Table S1. For a number of genes implicated in epileptic encephalopathy, future studies may provide more detailed associations with ASD features. Moreover, these numbers represent estimates rather than true prevalence rates because the primary emphasis of the source studies is not necessarily to characterize the ASD phenotype, which leads to indirect terminology such as “autistic features” rather than detailed accounts of formal ASD evaluations. Methodological limitations notwithstanding, there is ample evidence to suggest that within monogenic epileptic encephalopathies, a substantial percentage of patients may be affected by ASD characteristics.

### ASD features

Across monogenic epileptic encephalopathies, actual descriptions of core ASD features are sparse, but there are descriptions of various repetitive behaviors (see Additional file 1: Table S1). The spectrum of repetitive behaviors includes repetitive movements with a lower order cognitive component (such as motor stereotypies and self-injurious behavior [SIB]) as well as behaviors with a higher order cognitive component (such as obsessions and perseverations). In patients with epileptic encephalopathy and features of ASD, motor stereotypies are specifically noted in disorders involving the following genes: *CDKL5* (hand flapping) [32], *HCN1* [30], *IQSEC2* (hand flapping) [33], *MEF2C* (hand wringing) [34], *NRXN1* (body rocking) [35], *PCDH19* (hand flapping, hand mouthing, and spinning) [36], *SIK1* (hand flapping) [31], *SLC35A2* (head and hand stereotypies) [37], *SLC6A1* [29], and *STXBP1* (hand flapping and stereotyped hand washing) [38, 39]. Similarly, SIB occurs in *CDKL5*-related disorders (hand biting) [32], *IQSEC2*-related disorders [33], *PCDH19*-related disorders [36], and *SIK1*-related disorders (head banging and biting) [31], while obsessive/perseverative behaviors are associated with *PCDH19*-related disorders [36] and *SCN8A*-related disorders [40]. Though generally speaking the data is too limited to define an overarching ASD profile across multiple monogenic epileptic encephalopathies, description of different types of repetitive behaviors is certainly noteworthy.

### Related neurodevelopmental dysfunction

Defects in genes associated with epileptic encephalopathy and ASD can result in neurodevelopmental dysfunction beyond these two phenotypes. Attention-deficit hyperactivity disorder (ADHD)-like clinical characteristics can exist in individuals with ASD and epileptic encephalopathy who have alterations in *CHD2* [41], *GRIN2A* [26], *PCDH19* [36, 42], *SLC6A1* [29], and *STXBP1* [43]. Interestingly, some epileptic encephalopathy and ASD genes are separately implicated in psychiatric presentations: variants in *KCNQ2* [44, 45] and *KCNQ3* [46] may influence susceptibility to bipolar disorder (BPD), while alterations affecting *ERBB4* [47], *GRIN2A* [48], *GRIN2B* [49], *NRXN1* [49–54], *PCDH19* [55], *SCN2A* [56], *SLC12A5* [57], and *TCF4* [58, 59] may mediate risk for schizophrenia (SCZ). In addition, there is evidence to suggest that deletions involving *NRXN1* are implicated in Tourette syndrome [60], and variants in *GRIN2B* are associated with obsessive-compulsive disorder (OCD) [61]. ID is perhaps the most important factor to consider. Collectively, all of the genes underlying presentations of epileptic encephalopathy and ASD are associated with the full spectrum of ID.

It is intriguing that the same gene implicated in severe, intractable epilepsy may have other (and sometimes concomitant) childhood presentations which are more cognitive/behavioral in nature, such as ASD and ID, as well as adult-onset presentations which are more psychiatric in nature. This spectrum may reflect variable expressivity, which is commonly seen in epilepsy genetics [62]. Another possibility is that the type of variant may influence the disease course. Some genetic changes may confer susceptibility to a certain condition (e.g., SCZ), requiring interaction with other genetic and environmental factors before leading to the condition, while other variants may have a more direct impact on CNS pathology.

Given that there is considerable overlap between ID and ASD [63], one may argue that the ASD features seen in epileptic encephalopathy are not necessarily related to epilepsy but rather reflect the presence of ID. It could be that a genetic defect leads to severe to profound ID, which may be a substantial contributor to the appearance of an ASD-like phenotype, more so than either the genetic defect or epilepsy alone. In fact, with increasing ID severity, it becomes progressively difficult to disentangle a diagnosis of ASD from the social-communication deficits commensurate with a patient’s developmental age. Repetitive behaviors can occur with severe to profound ID, without necessarily reflecting the presence of ASD. However, when ID is severe to profound, one can acknowledge the presence of autism features but be careful about labeling a diagnosis of ASD. Moreover, there are also cases of mild and moderate ID in some monogenic epileptic encephalopathies, so one cannot discount completely the development of ASD in monogenic epileptic encephalopathies. In the following section, we will discuss the contribution of epilepsy to the cognitive/behavioral phenotype above and beyond what one would expect from the genetic defect alone.

## Impact of epilepsy on ASD in monogenic epileptic encephalopathies

### Key questions in the debate

Among epileptic encephalopathies, a critical and controversial subject is whether the features of epilepsy independently *worsen* the development of ASD. In other words, in a child with a genetic disorder associated with the presentation of epileptic encephalopathy, it is not clear if the seizures/interictal epileptiform activity serve as a so-called second-hit against an already vulnerable brain predisposed to developing epilepsy and ASD as separate downstream effects of a single gene defect [64]. Relevant to this central debate are the following auxiliary questions:

- Since IS are one of the most catastrophic forms of epilepsy, does their presence herald the onset of ASD related to increasingly disrupted brain function?
- With respect to other seizures types among monogenic epileptic encephalopathies, is seizure severity related to neurocognitive severity?
- Within epileptic encephalopathies associated with ASD, does the age of seizure onset predict neurodevelopmental outcomes? After all, if epileptic activity does in fact exacerbate the neurobehavioral phenotype, then the emergence of seizures at an earlier age—especially during the period of rapid brain growth that takes place in the first 2 years of life [65]—could portend worse outcomes.

In theory, the answers to all of these questions are yes based on the definition of epileptic encephalopathy according to the International League Against Epilepsy (ILEA) [4]; however, in reality, the evidence from clinical studies is mixed.

### Infantile spasms and ASD development

In cases of IS—one of the most common causes of epileptic encephalopathy—it is the underlying cause, more so than the spasms themselves, that helps predict the onset of ASD. IS are a distinct epilepsy syndrome with onset less than 1 year of age, appearing as clusters of flexion movements/extension movements/head drops, with a chaotic high-voltage EEG pattern called hypsarrhythmia (though atypical forms can exist without this pattern) [66]. In a heterogeneous cohort of 95 children with unprovoked, infantile-onset seizures comprising the basis for a case–control study, the diagnosis of ASD (which defined the cases) was not contingent on a history of IS after adjusting for whether the seizures were symptomatic, but it was associated with symptomatic seizures after adjusting for whether the seizures were IS. In other words, it is the presence of an underlying metabolic/genetic/structural cause, rather than the occurrence of IS per se, that more likely predicts ASD. In this study, ASD evaluation took place at a mean age of 11 years and 2 months, based on (1) meeting a certain cutoff on the Social Communication Questionnaire (SCQ), (2) subsequent evaluation with the Autism Diagnostic Interview-Revised (ADI-R), Autism Diagnostic Observation Schedule (ADOS), and Childhood Autism Rating Scale (CARS) suggesting impairments in the domains of social interaction, communication, and stereotyped/repetitive behavior in accordance with International Classification of Diseases-Tenth Revision (ICD-10) criteria. Out of the 13 cases (those with ASD), 6 had IS and 4 had a mental age < 24 months; out of the 82 controls (those without ASD), 11 had IS and 8 had a mental age < 24 months. In this study, ID and ASD were closely linked, as there was only one subject with ASD who did not have ID. However, when the analysis was limited to patients with a mental age >24 months, the odds ratio for ASD risk due to symptomatic seizures (and for ASD risk due to IS) actually increased, suggesting that ID severity was not the major driving force for ASD risk [67]. Likewise, out of the nine patients with IS who underwent a prospective treatment study involving either ACTH or vigabatrin with crossover for non-responders, the three individuals who developed ASD (based on the ADOS) all had symptomatic epilepsy. Two of the three were severely delayed in cognitive development on follow-up evaluations based on the Bayley Scales of Infant Development (the cognitive status of the third is unclear from the data). The other six patients in the cohort who did not develop ASD had, upon follow up assessment, variable cognitive outcomes, ranging from normal to significantly delayed development [68]. In a retrospective cohort of individuals with tuberous sclerosis complex (TSC)—a disorder of mTOR signaling caused by mutations in *TSC1* and *TSC2* and associated with IS and high prevalence rates of ASD (36%) [69]—IS occurrence predicted ASD development as determined by ICD-10 criteria in conjunction with review of ADI-R and ADOS testing [70]. However, in this study in the TSC population, not all individuals with IS developed ASD, and not all individuals with ASD had preceding IS [70]. Finally, in 90 children with epilepsy and ID stratified by ASD diagnosis (based on DSM-III-R and DSM-IV criteria), the presence of IS was not a distinguishing factor [71]. Taken together, these findings suggest that, from a mechanistic standpoint, ASD and IS may be two separate end-results of a common CNS defect.

Timely treatment of spasms does not necessarily impact the development of ASD. Among infants with new-onset IS treated rapidly with a standardized protocol and followed longitudinally, the incidence of ASD based on ADOS testing at >30 months was 23% [72]. The prevalence of ASD is 1.5% in the general population [73] and 9–35% in IS [67, 74–77], so this incidence of 23% falls within expected range, suggesting that early treatment of the spasms does not diminish ASD risk.

Children with IS who develop ASD may have persistent epileptiform abnormalities despite treatment of spasms. Such abnormalities can occur in the frontal [72] and temporal regions [72, 75]. Likewise, positron emission tomography (PET) studies show hypometabolism in these areas, in addition to the parietal lobe, in patients with IS who developed ASD [74, 78]. In some cases, epileptiform activity may precede onset of IS or epilepsy, such as with TSC [79, 80]. The time gap between detection of EEG abnormalities and onset of seizures or spasms in infants with TSC creates a window of opportunity for possible intervention. Indeed, there is an ongoing randomized, placebo-controlled clinical trial (Preventing Epilepsy Using Vigabatrin In Infants With Tuberous Sclerosis Complex; https://clinicaltrials.gov/ct2/show/NCT02849457) that will enroll infants with TSC in order to determine whether administration of vigabatrin before clinical seizure onset prevents epilepsy and improves neurobehavioral outcomes including ADOS scores. This treatment strategy may serve as a model for monogenic epileptic encephalopathies where ASD can be a downstream feature. Hence, in IS, while treatment of the spasms themselves may not prevent the development of ASD, treatment directed toward the preceding epileptic activity holds promise for altering this trajectory.

### Seizure severity versus neurocognitive severity

In cases of epileptic encephalopathy besides IS, epilepsy severity does not necessarily impact neurobehavioral outcomes. *STXBP1*-related encephalopathy is one example. *STXBP1* encodes syntaxin-binding protein 1, which helps regulate synaptic vesicle release [81]. Defects in this gene result in an epileptic encephalopathy [82] accompanied by ID, motor abnormalities (hypotonia, ataxia, tremor, spasticity, dyskinesia, dystonia), and ASD features [43]. Stamberger et al. [43] analyzed the relationship between cognitive outcomes and epilepsy severity in 147 patients with *STXBP1* encephalopathy, 95% of whom had epilepsy (including 21% who presented with OS, 9.5% who presented with West syndrome (WS), and 53% who presented as early-onset epilepsy and encephalopathy). The authors did not find a statistically significant relationship between level of cognitive impairment and either age at seizure onset or length of time from seizure onset to seizure freedom (ASD severity was not a reported outcome), though they did acknowledge that the study was underpowered. Furthermore, in this analysis, as noted above, about half the cases were early-onset epilepsy and encephalopathy, which is a different entity from epileptic encephalopathy [43].

DS is another example where cognitive outcomes do not necessarily reflect seizure severity. DS is characterized by myoclonic seizures in infancy, often associated with fever, that evolve into other seizure types [83]. Mutations in *SCN1A* (sodium voltage-gated channel alpha subunit 1)—which are implicated in sporadic cases of ASD [84]—are a common cause of DS [85]. In a prospective cohort of 67 patients with DS, developmental/intelligence quotient (DQ/IQ) did not correlate with measures of epilepsy severity [86]. Similar findings were present in a retrospective study of 21 patients with DS, ages 6–10 years [87]. These findings as a whole suggest that among epileptic encephalopathies, the underlying genetic defect may be the predominant contribution to the clinical presentation, overshadowing the possible impact of epilepsy severity on the cognitive phenotype. Of note, among monogenic epileptic encephalopathies, there has been a scarcity of studies that have contrasted the ASD phenotype to idiopathic ASD, especially in relation to epilepsy severity. Such studies will be essential to help delineate whether epilepsy severity impacts the ASD profile.

### Age of seizure onset related to ASD development

In contrast, the timing of ASD development—commonly after seizure onset in monogenic epileptic encephalopathies, most strikingly when regression is involved—argues *for* the notion that seizure activity may influence ASD emergence. One example is LKS, which results in language regression, characteristically in conjunction with temporal lobe epileptiform activity [25], and is linked to defects in *GRIN2A* [26, 88]. Along with regression and epileptic encephalopathy, features of ASD can occur in LKS [89]. Because the areas affected by epileptiform activity play a role in language, a prevailing thought is that the cognitive difficulties seen in this disorder are a direct consequence of electrographic changes. However, this hypothesis remains the subject of debate. It should be noted that the language regression associated with LKS is different from that of autistic regression. The former is characterized by relatively typical early development followed by language regression usually between 3–9 years of age [89]. In contrast, the latter is characterized by loss of language and social skills usually before 3 years of age, and early development may be typical or show subtle abnormalities [90].

Disruptions in other ion channel genes, including *SCN2A* and *SCN8A*, may also result in epileptic encephalopathy and developmental regression before emergence of ASD symptoms. One report describes a 29-year-old woman with a de novo nonsense variant in *SCN2A* who developed intractable epilepsy and cognitive decline starting with seizures at 1 year and 7 months of age as well as “autistic” and hyperkinetic features at 2 years of age [91]. *SCN2A* encodes one of the alpha subunits of the voltage-gated sodium channel and plays an important role in action potential generation and propagation (http://www.ncbi.nlm.nih.gov/gene/6326). Another report characterizes a female with a de novo *SCN8A* missense variant who developed generalized seizures at 6 months of age, followed by epileptic spasms at 4 years of age. Over the following 1–2 years, her language and socialization skills deteriorated while obsessive-compulsive and repetitive behaviors emerged, culminating in a diagnosis of ASD at 5 years of age [40].

Even without regression, other monogenic causes of epileptic encephalopathy also lead to ASD symptoms around the same time as, or after, seizure onset. *SCN2A* is not always associated with regression. A 10-year-old boy with a de novo splice site *SCN2A* variant had a presentation of ID, intractable epilepsy, hypotonia, intermittent ataxia, and cerebral/cerebellar brain atrophy. He developed seizures at 3 years of age, around the same time as his diagnosis of “autism” [92]. Out of six individuals with epileptic encephalopathies (early myoclonic encephalopathy, OS, and IS) due to *SIK1* variants, three developed features of ASD in the context of experiencing IS between 2–4 months of age [31]. In some instances, autism features can emerge in the wake of plateaued development and seizure onset, as is the case with *SCL35A2*-related disorders [37]. Overall, the data suggests that among these disorders, there could be a temporal relationship between seizure onset and ASD symptoms. However, the causative nature of this relationship is not fully clear, and larger, carefully phenotyped cohorts are necessary for further investigation. An alternative possibility is that in some cases, epileptic encephalopathy may indeed be contributing to emergence of ASD symptoms, while in others, epileptic encephalopathy and ASD may be two separate outcomes resulting from final common disrupted pathways due to the underlying gene defect. This mechanistic heterogeneity might reflect the vast range of cellular pathways implicated in different monogenic epileptic encephalopathies.

Furthermore, as discussed previously, it can be difficult to separate a diagnosis of ASD from severe ID as was the case for some of these examples that described level of cognitive impairment. Hence, one must be careful to characterize these as autistic features in the absence of careful evaluations of social-communication that take into account cognitive status.

## Genetic overlap between ASD and epileptic encephalopathy

### Studies linking epileptic encephalopathy genes to ASD

Despite uncertainty surrounding whether epileptic encephalopathy in and of itself drives ASD development, what is clear is that there is a rich genetic overlap between the two entities. Out of the 62 genes implicated in the development of epileptic encephalopathy (selected from [23]), 34 also serve as risk factors in the pathogenesis of ASD. This gene set includes causes of both idiopathic ASD (where ASD is the primary diagnosis) and syndromic ASD (where ASD exists alongside other clinical features that help define a recognized syndrome). The strongest evidence for ASD association, based on the Simons Foundation Autism Research Initiative Gene Scoring Criteria (https://gene.sfari.org/autdb/GS_Classification.do), comes in the form of full sequencing studies of phenotypically well-defined cohorts of individuals/families with ASD and unrelated controls. Such studies should demonstrate that recurrent pathogenic variants in certain genes occur at a statistically higher frequency in cases compared to controls and segregate with the ASD phenotype in families, if applicable; moreover, findings must be replicable.

Based on these criteria, *SCN2A*—commonly linked to epileptic encephalopathy—has the highest level of evidence supporting its role in ASD. It is one of the causes of a number of epileptic encephalopathy syndromes (early-onset epileptic encephalopathy, early infantile epileptic encephalopathy, IS, epilepsy of infancy with migrating focal seizures, and LGS) [23], and affected children can present with severe to profound ID, hypotonia, spasticity, movement disorders, and gastrointestinal symptoms [93]. Moreover, several studies have identified rare, likely pathogenic *SCN2A* variants in idiopathic ASD. Out of 117 multiplex ASD families who underwent *SCN1A*, *SCN2A*, and *SCN3A* sequencing, there was one affected individual with an *SCN2A* variant, which was absent in controls. Based on functional studies demonstrating disrupted calcium-calmodulin binding, this missense variant was characterized as a potential disease causing mutation [94]. In a whole-exome sequencing study of 238 simplex ASD families comparing probands to unaffected controls, de novo nonsense variants in *SCN2A* occurred in two of the probands, neither of whom had a history of seizures [95]. In a large-scale sequencing analysis of coding variants in ASD subjects versus controls involving a TADA (Transmission and De novo Association)-based statistical model, *SCN2A* had a false discovery rate of ≤0.01, suggesting a high likelihood for true association with ASD [96]. Overall, the data is convincing that *SCN2A* leads to neurodevelopmental dysfunction other than epileptic encephalopathy.

Multiple lines of evidence have highlighted the involvement of other epileptic encephalopathy genes, with diverse functions, in ASD (see Additional file 2: Table S2 and Additional file 3: Table S3). We have organized evidence connecting each of these genes to ASD into the following three categories: (1) variant discovery (next-generation sequencing, single nucleotide polymorphism (SNP) association study, and copy number variant analysis) in cohorts where ASD is the primary phenotype; (2) variant discovery (next-generation sequencing) in cohorts with ASD-related neurodevelopmental phenotypes (e.g., epilepsy and ID), revealing genetic findings in a subset of patients who also have ASD; and (3) clinical characterization (case reports/series) of known genetic disorders in patients, some of whom may have ASD as a manifestation of the disease. Focusing on well-defined ASD cohorts, whole-exome or targeted sequencing studies have implicated genes involved in ion channel function (*GABRA1*, *GABRB1*, *GRIN2A*, *KCNQ2*, *KCNQ3*), ion transporter function (*SLC6A1*, *SLC12A5*), transcriptional regulation (*ARX*, *TCF4*), signal transduction (*SIK1*), cell adhesion (*PCDH19*), and DNA replication (*SETBP1*). A small number of SNP and CNV studies have additionally connected ASD to variants in *ERBB4, FLNA, GABRG2,* and *PLCB1,* while descriptive case series on patients with variants in a specific gene of interest have revealed ASD associations with *CACNA1A, GRIN1*, *HCN1*, *IQSEC2*, *SCN8A*, and *SLC35A2*.

An important caveat is that many of the case reports/case series in Additional file 2: Table S2 which referenced ASD defined it poorly or nonspecifically. In these instances, affected individuals did not have a formal diagnosis of ASD based on, for example, DSM criteria. Rather, they had “autistic features” or a label of ASD without a clear indication of diagnostic methodology or further delineation of the behavioral phenotype. Nonetheless, given the relatively rare occurrence of genetic epileptic encephalopathies, these studies are worth noting until larger cohorts of affected patients become available for rigorous neurodevelopmental phenotyping.

## Common final pathways shared between ASD and epileptic encephalopathy

### Overview

The genetic diversity surrounding monogenic epileptic encephalopathies associated with ASD suggests multiple possible mechanisms for epileptogenesis and cognitive/behavioral disturbance. Collectively, these genes play fundamental roles in human neurobiology, including synapse formation, action potential generation, and neurotransmitter release. It is easy to imagine how disruption to any one of these processes can lead to CNS dysfunction through independent mechanisms related to the specific gene function. However, in order to explain clinical entities like ASD and epileptic encephalopathy in the context of multiple single-gene etiologies, it may be reasonable to consider that these phenotypes represent the end-product of final common disrupted pathways. Below, we will discuss one such pathway that serves as possible explanations for both ASD and epileptic encephalopathy in the setting of shared monogenic etiologies. This constitutes a salient example rather than an exhaustive list.

### Disrupted excitatory inhibitory balance

Features of ASD and epileptic encephalopathy may result from an imbalance between excitatory and inhibitory signaling in the CNS. The list of genes implicated in ASD and epileptic encephalopathy includes several that are involved in the function of ion channels that play important roles in the brain, like voltage-gated sodium channels or γ-aminobutyric acid (GABA) type A (GABA_A_) receptors. Increased excitatory neurotransmission (especially from sodium channel alterations) and decreased inhibitory neurotransmission (especially from GABA_A_ receptor alterations) both result in neuronal hyperexcitability, one of the hallmarks of epilepsy [97]. Furthermore, GABAergic interneurons are instrumental in shaping neuronal circuits through various mechanisms, with implications for brain development and neuronal plasticity [98, 99]. Therefore, defects in GABAergic signaling—specifically tilting the excitatory/inhibitory balance towards excitatory signaling—may explain some of the features of ASD [100].

Further evidence for the role of GABAergic signaling in cognition, behavior, and epileptogenesis comes from study of two neurodevelopmental disorders, Prader-Willi syndrome (PWS) and Angelman syndrome (AS). These are disorders that both involve inactivation or deletion of one parent’s contribution to chromosome 15q11.2-q13, but they result in different phenotypes because of parent-specific imprinting patterns leaving certain genes epigenetically silenced. AS is characterized by severe to profound ID, epilepsy, and ataxia, while PWS is characterized by mild intellectual disability, behavioral problems, and endocrine abnormalities, among other features. Both feature a relatively high prevalence of ASD [101]. About 5% of cases of AS and 25% of cases of PWS result from uniparental disomy (UPD), while a significantly larger fraction (70%) stem from chromosomal deletions in 15q11.2-q13 [102]. This chromosomal segment contains both imprinted and non-imprinted genes, and among the non-imprinted genes are *GABRA5*, *GABRB3*, and *GABRG3* [103]. PWS and AS patients with chromosome 15q deletions have fewer working copies of these non-imprinted genes compared to PWS and AS patients with UPD. Interestingly, patients in the former category are also more affected than those in the latter category, with poorer physical growth, more frequent seizures, higher incidence of microcephaly, more ataxia, and lower cognitive skills in the case of AS [104] and more frequent seizures in the case of PWS [105]. Taken together, these findings underscore the crucial role of GABAergic signaling and excitatory-inhibitory balance in neurodevelopmental functioning ranging from seizure threshold to behavior and cognition, which serves as but one illustrative example of final common pathways shared between epilepsy and ASD development.

## Conclusions

In summary, there is phenotypic overlap in the presentations of epileptic encephalopathy and ASD. Certain types of epileptic encephalopathy are more commonly associated with development of ASD, including IS. However, the precise relationship between epileptic encephalopathy and ASD remains the subject of continued discovery. Finally, advances in genetics have revealed genes that overlap in ties to both ASD and epileptic encephalopathy. Overall, many unanswered questions remain about the connection between ASD and monogenic epileptic encephalopathies, including how to differentiate severe to profound ID from ASD—a common and well-known challenge across many genetic and non-genetic conditions. Nonetheless, clinicians caring for children with epileptic encephalopathies should be aware of at least the possibility of the emergence of ASD in conjunction with other developmental delays. Given the strong likelihood of developmental impairment associated with monogenic epileptic encephalopathies, developmental surveillance is crucial starting from infancy. In fact, statewide programs providing early intervention services often allow eligibility not only for infants and toddlers with established developmental diagnoses but also for those who have medical and genetic conditions associated with a high risk of future developmental disabilities (though exact statutes may vary from state to state). Epileptologists and other clinicians taking care of patients with epileptic encephalopathies can facilitate referrals to these kinds of services or to developmental pediatricians. If a diagnosis of ASD is made, then early intervention with applied behavioral analysis therapy may be helpful [105].

## Additional files

Additional file 1: Table S1. ASD and epilepsy features associated with monogenic epileptic encephalopathies. (DOCX 93 kb)

Additional file 2: Table S2. Evidence linking epileptic encephalopathy genes to ASD. (DOCX 218 kb)

Additional file 3: Table S3. Functions of epileptic encephalopathy genes linked to ASD. (DOCX 19 kb)

## Abbreviations

ADI-R: Autism Diagnostic Interview-Revised
ADOS: Autism Diagnostic Observation Schedule
AS: Angelman syndrome
ASD: Autism spectrum disorder
BPD: Bipolar disorder
CARS: Childhood Autism Rating Scale
CNS: Central nervous system
CNV: Copy number variation
CSWSS: Continuous spike-wave discharges in slow wave sleep syndrome
DQ/IQ: Developmental/intelligence quotient
DS: Dravet syndrome
DSM: Diagnostic and Statistical Manual of Mental Disorders
GABA: γ-Aminobutyric acid
ICD: International Classification of Diseases
ID: Intellectual disability
ILEA: International League Against Epilepsy
IS: Infantile spasms
LGS: Lennox–Gastaut syndrome
LKS: Landau-Kleffner syndrome
OCD: Obsessive-compulsive disorder
OS: Ohtahara syndrome
PET: Positron emission tomography
PWS: Prader-Willi syndrome
SCQ: Social Communication Questionnaire
SCZ: Schizophrenia
SIB: Self-injurious behavior
SNP: Single nucleotide polymorphism
TSC: Tuberous sclerosis complex
WS: West syndrome

## References

[1] American Psychiatric Association, American Psychiatric Association, DSM-5 Task Force (2013). Diagnostic and statistical manual of mental disorders: DSM-5.

[2] Bolton PF, Carcani-Rathwell I, Hutton J, Goode S, Howlin P, Rutter M (2011). Epilepsy in autism: features and correlates. Br J Psychiatry J Ment Sci.

[3] Thomas S, Hovinga ME, Rai D, Lee BK. Brief report: prevalence of co-occurring epilepsy and autism spectrum disorder: the U.S. National Survey of Children’s Health 2011–2012. J Autism Dev Disord. 2016. 10.1007/s10803-016-2938-7 27752862

[4] Berg AT, Berkovic SF, Brodie MJ, Buchhalter J, Cross JH, van Emde Boas W (2010). Revised terminology and concepts for organization of seizures and epilepsies: report of the ILAE Commission on Classification and Terminology, 2005–2009. Epilepsia.

[5] Marsh ED, Brooks-Kayal AR, Porter BE (2006). Seizures and antiepileptic drugs: does exposure alter normal brain development?. Epilepsia.

[6] de la Torre-Ubieta L, Won H, Stein JL, Geschwind DH (2016). Advancing the understanding of autism disease mechanisms through genetics. Nat Med.

[7] Abrahams BS, Geschwind DH (2008). Advances in autism genetics: on the threshold of a new neurobiology. Nat Rev Genet.

[8] Loke YJ, Hannan AJ, Craig JM (2015). The role of epigenetic change in autism spectrum disorders. Front Neurol.

[9] Bailey A, Le Couteur A, Gottesman I, Bolton P, Simonoff E, Yuzda E (1995). Autism as a strongly genetic disorder: evidence from a British twin study. Psychol Med.

[10] Steffenburg S, Gillberg C, Hellgren L, Andersson L, Gillberg IC, Jakobsson G (1989). A twin study of autism in Denmark, Finland, Iceland, Norway and Sweden. J Child Psychol Psychiatry.

[11] Jorde LB, Hasstedt SJ, Ritvo ER, Mason-Brothers A, Freeman BJ, Pingree C (1991). Complex segregation analysis of autism. Am J Hum Genet.

[12] Bishop DVM, Maybery M, Maley A, Wong D, Hill W, Hallmayer J (2004). Using self-report to identify the broad phenotype in parents of children with autistic spectrum disorders: a study using the Autism-Spectrum Quotient. J Child Psychol Psychiatry.

[13] Bolton P, Macdonald H, Pickles A, Rios P, Goode S, Crowson M (1994). A case–control family history study of autism. J Child Psychol Psychiatry.

[14] Weiss LA, Shen Y, Korn JM, Arking DE, Miller DT, Fossdal R (2008). Association between microdeletion and microduplication at 16p11.2 and autism. N Engl J Med.

[15] Urraca N, Cleary J, Brewer V, Pivnick EK, McVicar K, Thibert RL (2013). The interstitial duplication 15q11.2-q13 syndrome includes autism, mild facial anomalies and a characteristic EEG signature. Autism Res Off J Int Soc Autism Res.

[16] Prasad A, Merico D, Thiruvahindrapuram B, Wei J, Lionel AC, Sato D (2012). A discovery resource of rare copy number variations in individuals with autism spectrum disorder. G3 Bethesda Md.

[17] Zhao X, Leotta A, Kustanovich V, Lajonchere C, Geschwind DH, Law K (2007). A unified genetic theory for sporadic and inherited autism. Proc Natl Acad Sci U S A.

[18] Bourgeron T (2015). From the genetic architecture to synaptic plasticity in autism spectrum disorder. Nat Rev Neurosci.

[19] Allen AS, Berkovic SF, Cossette P, Delanty N, Epi4K Consortium, Epilepsy Phenome/Genome Project (2013). De novo mutations in epileptic encephalopathies. Nature.

[20] Michaud JL, Lachance M, Hamdan FF, Carmant L, Lortie A, Diadori P (2014). The genetic landscape of infantile spasms. Hum Mol Genet.

[21] Jeste SS, Tuchman R (2015). Autism spectrum disorder and epilepsy: two sides of the same coin?. J Child Neurol.

[22] El Achkar CM, Spence SJ (2015). Clinical characteristics of children and young adults with co-occurring autism spectrum disorder and epilepsy. Epilepsy Behav EB.

[23] McTague A, Howell KB, Cross JH, Kurian MA, Scheffer IE (2016). The genetic landscape of the epileptic encephalopathies of infancy and childhood. Lancet Neurol.

[24] Ramanathan RS, Ahluwalia T, Sharma A (2010). Lennox-Gastaut syndrome: an overview. J Pediatr Neurosci.

[25] Tassinari CA, Rubboli G, Volpi L, Meletti S, d’Orsi G, Franca M (2000). Encephalopathy with electrical status epilepticus during slow sleep or ESES syndrome including the acquired aphasia. Clin Neurophysiol Off J Int Fed Clin Neurophysiol.

[26] Lesca G, Rudolf G, Bruneau N, Lozovaya N, Labalme A, Boutry-Kryza N (2013). GRIN2A mutations in acquired epileptic aphasia and related childhood focal epilepsies and encephalopathies with speech and language dysfunction. Nat Genet.

[27] Zhao Y, Zhang X, Bao X, Zhang Q, Zhang J, Cao G (2014). Clinical features and gene mutational spectrum of CDKL5-related diseases in a cohort of Chinese patients. BMC Med Genet.

[28] Kwong AK-Y, Fung C-W, Chan S-Y, Wong VC-N (2012). Identification of SCN1A and PCDH19 mutations in Chinese children with Dravet syndrome. PLoS One.

[29] Carvill GL, McMahon JM, Schneider A, Zemel M, Myers CT, Saykally J (2015). Mutations in the GABA transporter SLC6A1 cause epilepsy with myoclonic-atonic seizures. Am J Hum Genet.

[30] Nava C, Dalle C, Rastetter A, Striano P, de Kovel CGF, Nabbout R (2014). De novo mutations in HCN1 cause early infantile epileptic encephalopathy. Nat Genet.

[31] Hansen J, Snow C, Tuttle E, Ghoneim DH, Yang C-S, Spencer A (2015). De novo mutations in SIK1 cause a spectrum of developmental epilepsies. Am J Hum Genet.

[32] Russo S, Marchi M, Cogliati F, Bonati MT, Pintaudi M, Veneselli E (2009). Novel mutations in the CDKL5 gene, predicted effects and associated phenotypes. Neurogenetics.

[33] Gandomi SK, Farwell Gonzalez KD, Parra M, Shahmirzadi L, Mancuso J, Pichurin P (2014). Diagnostic exome sequencing identifies two novel IQSEC2 mutations associated with X-linked intellectual disability with seizures: implications for genetic counseling and clinical diagnosis. J Genet Couns.

[34] Paciorkowski AR, Traylor RN, Rosenfeld JA, Hoover JM, Harris CJ, Winter S (2013). MEF2C Haploinsufficiency features consistent hyperkinesis, variable epilepsy, and has a role in dorsal and ventral neuronal developmental pathways. Neurogenetics.

[35] Duong L, Klitten LL, Møller RS, Ingason A, Jakobsen KD, Skjødt C (2012). Mutations in NRXN1 in a family multiply affected with brain disorders: NRXN1 mutations and brain disorders. Am J Med Genet Part B Neuropsychiatr Genet Off Publ Int Soc Psychiatr Genet.

[36] Jamal SM, Basran RK, Newton S, Wang Z, Milunsky JM (2010). Novel de novo PCDH19 mutations in three unrelated females with epilepsy female restricted mental retardation syndrome. Am J Med Genet A.

[37] Lopes F, Barbosa M, Ameur A, Soares G, de Sá J, Dias AI, et al. Identification of novel genetic causes of Rett syndrome-like phenotypes. J Med Genet. 2016;53(3):190-9, 10.1136/jmedgenet-2015-103568 26740508

[38] Romaniello R, Saettini F, Panzeri E, Arrigoni F, Bassi MT, Borgatti R (2015). A de-novo STXBP1 gene mutation in a patient showing the Rett syndrome phenotype. Neuroreport.

[39] Deciphering Developmental Disorders Study (2015). Large-scale discovery of novel genetic causes of developmental disorders. Nature.

[40] Veeramah KR, O’Brien JE, Meisler MH, Cheng X, Dib-Hajj SD, Waxman SG (2012). De novo pathogenic SCN8A mutation identified by whole-genome sequencing of a family quartet affected by infantile epileptic encephalopathy and SUDEP. Am J Hum Genet.

[41] Suls A, Jaehn JA, Kecskés A, Weber Y, Weckhuysen S, Craiu DC (2013). De novo loss-of-function mutations in CHD2 cause a fever-sensitive myoclonic epileptic encephalopathy sharing features with Dravet syndrome. Am J Hum Genet.

[42] van Harssel JJT, Weckhuysen S, van Kempen MJA, Hardies K, Verbeek NE, de Kovel CGF (2013). Clinical and genetic aspects of PCDH19-related epilepsy syndromes and the possible role of PCDH19 mutations in males with autism spectrum disorders. Neurogenetics.

[43] Stamberger H, Nikanorova M, Willemsen MH, Accorsi P, Angriman M, Baier H (2016). STXBP1 encephalopathy: a neurodevelopmental disorder including epilepsy. Neurology.

[44] Borsotto M, Cavarec L, Bouillot M, Romey G, Macciardi F, Delaye A (2007). PP2A-Bgamma subunit and KCNQ2 K+ channels in bipolar disorder. Pharmacogenomics J.

[45] Judy JT, Seifuddin F, Pirooznia M, Mahon PB, Jancic D, Bipolar Genome Study Consortium (2013). Converging evidence for epistasis between ANK3 and potassium channel gene KCNQ2 in bipolar disorder. Front Genet.

[46] Zhang P, Xiang N, Chen Y, Sliwerska E, McInnis MG, Burmeister M (2010). Family-based association analysis to finemap bipolar linkage peak on chromosome 8q24 using 2,500 genotyped SNPs and 15,000 imputed SNPs. Bipolar Disord.

[47] Walsh T, McClellan JM, McCarthy SE, Addington AM, Pierce SB, Cooper GM (2008). Rare structural variants disrupt multiple genes in neurodevelopmental pathways in schizophrenia. Science.

[48] Tarabeux J, Kebir O, Gauthier J, Hamdan FF, Xiong L, Piton A (2011). Rare mutations in N-methyl-D-aspartate glutamate receptors in autism spectrum disorders and schizophrenia. Transl Psychiatry.

[49] Awadalla P, Gauthier J, Myers RA, Casals F, Hamdan FF, Griffing AR (2010). Direct measure of the de novo mutation rate in autism and schizophrenia cohorts. Am J Hum Genet.

[50] Vrijenhoek T, Buizer-Voskamp JE, van der Stelt I, Strengman E (2008). Genetic Risk and Outcome in Psychosis (GROUP) Consortium, Sabatti C, et al. Recurrent CNVs disrupt three candidate genes in schizophrenia patients. Am J Hum Genet.

[51] Kong A, Frigge ML, Masson G, Besenbacher S, Sulem P, Magnusson G (2012). Rate of de novo mutations and the importance of father’s age to disease risk. Nature.

[52] Gauthier J, Siddiqui TJ, Huashan P, Yokomaku D, Hamdan FF, Champagne N (2011). Truncating mutations in NRXN2 and NRXN1 in autism spectrum disorders and schizophrenia. Hum Genet.

[53] Hu X, Zhang B, Liu W, Paciga S, He W, Lanz TA (2014). A survey of rare coding variants in candidate genes in schizophrenia by deep sequencing. Mol Psychiatry.

[54] Rujescu D, Ingason A, Cichon S, Pietiläinen OPH, Barnes MR, Toulopoulou T (2009). Disruption of the neurexin 1 gene is associated with schizophrenia. Hum Mol Genet.

[55] Piton A, Gauthier J, Hamdan FF, Lafrenière RG, Yang Y, Henrion E (2011). Systematic resequencing of X-chromosome synaptic genes in autism spectrum disorder and schizophrenia. Mol Psychiatry.

[56] Li J, Cai T, Jiang Y, Chen H, He X, Chen C (2016). Genes with de novo mutations are shared by four neuropsychiatric disorders discovered from NPdenovo database. Mol Psychiatry.

[57] Merner ND, Chandler MR, Bourassa C, Liang B, Khanna AR, Dion P (2015). Regulatory domain or CpG site variation in SLC12A5, encoding the chloride transporter KCC2, in human autism and schizophrenia. Front Cell Neurosci.

[58] Steinberg S, de Jong S (2011). Irish Schizophrenia Genomics Consortium, Andreassen OA, Werge T, Børglum AD, et al. Common variants at VRK2 and TCF4 conferring risk of schizophrenia. Hum. Mol Genet.

[59] Stefansson H, Ophoff RA, Steinberg S, Andreassen OA, Cichon S, Rujescu D (2009). Common variants conferring risk of schizophrenia. Nature.

[60] Nag A, Bochukova EG, Kremeyer B, Campbell DD, Muller H, Valencia-Duarte AV (2013). CNV analysis in Tourette syndrome implicates large genomic rearrangements in COL8A1 and NRXN1. PLoS One.

[61] Arnold PD, Rosenberg DR, Mundo E, Tharmalingam S, Kennedy JL, Richter MA (2004). Association of a glutamate (NMDA) subunit receptor gene (GRIN2B) with obsessive-compulsive disorder: a preliminary study. Psychopharmacology (Berl).

[62] Olson HE, Poduri A, Pearl PL (2014). Genetic forms of epilepsies and other paroxysmal disorders. Semin Neurol.

[63] Polyak A, Kubina RM, Girirajan S (2015). Comorbidity of intellectual disability confounds ascertainment of autism: implications for genetic diagnosis. Am J Med Genet Part B Neuropsychiatr Genet Off Publ Int Soc Psychiatr Genet.

[64] Tuchman R (2006). Autism and epilepsy: what has regression got to do with it?. Epilepsy Curr Am Epilepsy Soc.

[65] Knickmeyer RC, Gouttard S, Kang C, Evans D, Wilber K, Smith JK (2008). A structural MRI study of human brain development from birth to 2 years. J Neurosci Off J Soc Neurosci.

[66] Shields WD (2006). Infantile spasms: little seizures. BIG consequences Epilepsy Curr.

[67] Saemundsen E, Ludvigsson P, Rafnsson V (2008). Risk of autism spectrum disorders after infantile spasms: a population-based study nested in a cohort with seizures in the first year of life. Epilepsia.

[68] Askalan R, Mackay M, Brian J, Otsubo H, McDermott C, Bryson S (2003). Prospective preliminary analysis of the development of autism and epilepsy in children with infantile spasms. J Child Neurol.

[69] Richards C, Jones C, Groves L, Moss J, Oliver C (2015). Prevalence of autism spectrum disorder phenomenology in genetic disorders: a systematic review and meta-analysis. Lancet Psychiatry.

[70] Bolton PF, Park RJ, Higgins JNP, Griffiths PD, Pickles A (2002). Neuro-epileptic determinants of autism spectrum disorders in tuberous sclerosis complex. Brain J Neurol.

[71] Steffenburg S, Steffenburg U, Gillberg C (2003). Autism spectrum disorders in children with active epilepsy and learning disability: comorbidity, pre- and perinatal background, and seizure characteristics. Dev Med Child Neurol.

[72] Bitton JY, Demos M, Elkouby K, Connolly M, Weiss SK, Donner EJ (2015). Does treatment have an impact on incidence and risk factors for autism spectrum disorders in children with infantile spasms?. Epilepsia.

[73] Christensen DL, Baio J, Van Naarden Braun K, Bilder D, Charles J, Constantino JN (2016). Prevalence and characteristics of autism spectrum disorder among children aged 8 years—autism and developmental disabilities monitoring network, 11 Sites, United States, 2012. Morb Mortal Wkly Rep Surveill Summ Wash DC 2002.

[74] Dilber C, Calışkan M, Sönmezoğlu K, Nişli S, Mukaddes NM, Tatlı B (2013). Positron emission tomography findings in children with infantile spasms and autism. J Clin Neurosci Off J Neurosurg Soc Australas.

[75] Riikonen R, Amnell G (1981). Psychiatric disorders in children with earlier infantile spasms. Dev Med Child Neurol.

[76] Saemundsen E, Ludvigsson P, Rafnsson V (2007). Autism spectrum disorders in children with a history of infantile spasms: a population-based study. J Child Neurol.

[77] Sidenvall R, Eeg-Olofsson O (1995). Epidemiology of infantile spasms in Sweden. Epilepsia.

[78] Chugani HT, Da Silva E, Chugani DC (1996). Infantile spasms: III. Prognostic implications of bitemporal hypometabolism on positron emission tomography. Ann Neurol.

[79] Wu JY, Peters JM, Goyal M, Krueger D, Sahin M, Northrup H (2016). Clinical electroencephalographic biomarker for impending epilepsy in asymptomatic tuberous sclerosis complex infants. Pediatr Neurol.

[80] Domańska-Pakieła D, Kaczorowska M, Jurkiewicz E, Kotulska K, Dunin-Wąsowicz D, Jóźwiak S (2014). EEG abnormalities preceding the epilepsy onset in tuberous sclerosis complex patients—a prospective study of 5 patients. Eur J Paediatr Neurol EJPN Off J Eur Paediatr Neurol Soc.

[81] Verhage M, Maia AS, Plomp JJ, Brussaard AB, Heeroma JH, Vermeer H (2000). Synaptic assembly of the brain in the absence of neurotransmitter secretion. Science.

[82] Saitsu H, Kato M, Mizuguchi T, Hamada K, Osaka H, Tohyama J (2008). De novo mutations in the gene encoding STXBP1 (MUNC18-1) cause early infantile epileptic encephalopathy. Nat Genet.

[83] Millichap JJ, Koh S, Laux LC, Nordli DR (2009). Child neurology: Dravet syndrome. Neurology.

[84] O’Roak BJ, Deriziotis P, Lee C, Vives L, Schwartz JJ, Girirajan S (2011). Exome sequencing in sporadic autism spectrum disorders identifies severe de novo mutations. Nat Genet.

[85] Claes L, Del-Favero J, Ceulemans B, Lagae L, Van Broeckhoven C, De Jonghe P (2001). De novo mutations in the sodium-channel gene SCN1A cause severe myoclonic epilepsy of infancy. Am J Hum Genet.

[86] Nabbout R, Chemaly N, Chipaux M, Barcia G, Bouis C, Dubouch C (2013). Encephalopathy in children with Dravet syndrome is not a pure consequence of epilepsy. Orphanet J Rare Dis.

[87] Villeneuve N, Laguitton V, Viellard M, Lépine A, Chabrol B, Dravet C (2014). Cognitive and adaptive evaluation of 21 consecutive patients with Dravet syndrome. Epilepsy Behav EB.

[88] Carvill GL, Regan BM, Yendle SC, O’Roak BJ, Lozovaya N, Bruneau N (2013). GRIN2A mutations cause epilepsy-aphasia spectrum disorders. Nat Genet.

[89] Pearl PL, Carrazana EJ, Holmes GL (2001). The Landau-Kleffner syndrome. Epilepsy Curr.

[90] Rogers SJ (2004). Developmental regression in autism spectrum disorders. Ment Retard Dev Disabil Res Rev.

[91] Kamiya K, Kaneda M, Sugawara T, Mazaki E, Okamura N, Montal M (2004). A nonsense mutation of the sodium channel gene SCN2A in a patient with intractable epilepsy and mental decline. J Neurosci Off J Soc Neurosci.

[92] Howell KB, McMahon JM, Carvill GL, Tambunan D, Mackay MT, Rodriguez-Casero V (2015). SCN2A encephalopathy: A major cause of epilepsy of infancy with migrating focal seizures. Neurology.

[93] Weiss LA, Escayg A, Kearney JA, Trudeau M, MacDonald BT, Mori M (2003). Sodium channels SCN1A, SCN2A and SCN3A in familial autism. Mol Psychiatry.

[94] Sanders SJ, Murtha MT, Gupta AR, Murdoch JD, Raubeson MJ, Willsey AJ (2012). De novo mutations revealed by whole-exome sequencing are strongly associated with autism. Nature.

[95] De Rubeis S, He X, Goldberg AP, Poultney CS, Samocha K, Cicek AE (2014). Synaptic, transcriptional and chromatin genes disrupted in autism. Nature.

[96] Bromfield EB, Cavazos JE, Sirven JI. Basic mechanisms underlying seizures and epilepsy [Internet]. American Epilepsy Society; 2006 [cited 2016 Aug 11]. Available from: http://www.ncbi.nlm.nih.gov/books/NBK2510/

[97] Möhler H (2007). Molecular regulation of cognitive functions and developmental plasticity: impact of GABAA receptors. J Neurochem.

[98] Gogolla N, Leblanc JJ, Quast KB, Südhof TC, Fagiolini M, Hensch TK (2009). Common circuit defect of excitatory-inhibitory balance in mouse models of autism. J Neurodev Disord.

[99] Pizzarelli R, Cherubini E (2011). Alterations of GABAergic signaling in autism spectrum disorders. Neural Plast.

[100] Zafeiriou DI, Ververi A, Dafoulis V, Kalyva E, Vargiami E (2013). Autism spectrum disorders: the quest for genetic syndromes. Am J Med Genet Part B Neuropsychiatr Genet Off Publ Int Soc Psychiatr Genet.

[101] Cassidy SB, Dykens E, Williams CA (2000). Prader-Willi and Angelman syndromes: sister imprinted disorders. Am J Med Genet.

[102] Hogart A, Wu D, LaSalle JM, Schanen NC (2010). The comorbidity of autism with the genomic disorders of chromosome 15q11.2-q13. Neurobiol Dis.

[103] Varela MC, Kok F, Otto PA, Koiffmann CP (2004). Phenotypic variability in Angelman syndrome: comparison among different deletion classes and between deletion and UPD subjects. Eur J Hum Genet EJHG.

[104] Varela MC, Kok F, Setian N, Kim CA, Koiffmann CP (2005). Impact of molecular mechanisms, including deletion size, on Prader-Willi syndrome phenotype: study of 75 patients. Clin Genet.

[105] Weitlauf AS, McPheeters ML, Peters B, Sathe N, Travis R, Aiello R, et al. Therapies for children with autism spectrum disorder: behavioral interventions update [Internet]. Rockville (MD): Agency for Healthcare Research and Quality (US); 2014 [cited 2017 Feb 25]. Available from: http://www.ncbi.nlm.nih.gov/books/NBK241444/25210724

