# Supplementary material for: Autism spectrum disorder and epileptic encephalopathy: common causes, many questions
Source: J Neurodev Disord. 2017 Jun 23;9:23. doi: 10.1186/s11689-017-9202-0 (PMC5481888; doi:10.1186/s11689-017-9202-0)
Supplement: Supplementary file 1 — ASD and epilepsy features associated with monogenic epileptic encephalopathies. (DOCX 93 kb) [file 11689_2017_9202_MOESM1_ESM.docx]

**Table S1. ASD and epilepsy features associated with monogenic epileptic encephalopathies.**

| **Gene** | **Associated Epileptic Encephalopathy Syndromes** | **Cases with ASD Features and Epileptic Encephalopathy** | **Reported Age of Seizure Onset in Cases with ASD Features and Epileptic Encephalopathy** | **Types of Seizures Reported in Cases with ASD Features and Epileptic Encephalopathy** | **Reported Description of ASD Features in Cases with ASD Features and Epileptic Encephalopathy** | **Prevalence of ASD Features in Cases with Epileptic Encephalopathy** | **Regression Reported in Cases with ASD Features and Epileptic Encephalopathy** | **Range of ID Reported in Cases with ASD Features and Epileptic Encephalopathy** | **Other Neurobehavioral Features Reported in Cases of ASD Features and Epileptic Encephalopathy** |
| --- | --- | --- | --- | --- | --- | --- | --- | --- | --- |
| *ARX* | EIEE, EOEE, IS | n=1 w/ “autistic/psychotic” features, IS [1] | 3m[1] | IS[1] | -routine dependent[1]  -limited eye contact[1]  -few friends[1] |  | yes[1] | severe[1] |  |
| *CDKL5* | IS, LGS | n=1 w/ WS, “autism”[2]  n=10 w/ “autistic features”, epileptic encephalopathy[3]  n=2 w/ epileptic encephalopathy, “autistic characteristics”[4]  n=1 w/ intractable epilepsy, “autistic” features[5] | 10d-3.3m [2–5] | FS [3], FSIC[5], GTCS[5], IS[2,3], MS[3,5], TS[3] | -avoidant eye contact[3,5]  -hand flapping [5]  -other hand stereotypies [2]  -SIB [5] | 100% (10/10 with “autistic features”)[3] |  | severe[2–5] | -mood lability[2] |
| *CHD2* | EMAS, LGS | n=1 w/ “ASD”, DS/epileptic encephalopathy[6]  n=1 w/ “ASD”, epileptic encephalopathy[7] | 2.5y-3.5y[6,7] | AAS[6], Abs[7], AS[6], FeS[6], FSIC[7], GTCS[6], HS[6], MS[6,7], TS[7] |  | 16.7% (1/6 w/ “ASD”) [7] |  | mild-severe[6,7] | -ADHD[6] |
| *GRIN1* | IS | n=1 w/ “ASD”, intractable epilepsy [8] | 22m[8] | FSIC, GTCS, GTS, GeS, SE, TAbS[8] | -high pain threshold[8] | 14.3% (1/7 w/ “ASD”) [8] |  | severe[8] |  |
| *GRIN2A* | EAS, IS | n=2 w/ “autistic features”, CSWSS[9] | 2y-4y[9] | CSWSS[9], FSIC[9], GTCS[9] |  | 5.6% (2/36 w/ “autistic features”) [9] | yes[9] |  | -ADHD features[9] |
| *GRIN2B* | IS, LGS | n=1 w/ “ASD”, LGS[10] | 6y[10] | AbS[10] |  |  |  |  |  |
| *HCN1* | DS | n=4 w/ “autistic features”, EIEE[11] | 4m-10m[11] | AAS[11], AS[11], CS[11], FeS[11], FS[11], HS[11], MS[11], NFS[11], TCS[11] | -stereotypies[11] | 67% (4/6 w/ “autistic features”)[11] |  | moderate-severe[11] | -aggression[11]  -agitation[11] |
| *IQSEC2* | IS | n=2 w/ “autistic-like features”, intractable epilepsy[12] | 2y[12] | MS[12] | -absent desire for social relationships[12]  -absent response to name[12]  -SIB[12]  -stereotypies (hand flapping)[12] |  |  |  |  |
| *KCNQ2* | EIEE, EOEE | n=1 w/ “autistic features”, EOEE[13] | neonatal period[13] | FSSG[13], GTCS[13], TS[13] |  | 6.3% (1/16 w/ “autistic features”)[13] |  |  |  |
| *MEF2C* | IS | n=1 w/ “ASD”, epileptic encephalopathy [7]  n=1 w/ “autism plus” phenotype, EMAS [14] | 11m-13m[7,14] | AbS[7], FeS[7,14], MAS[14], SE[7], TCS[7] | -stereotypies (hand-wringing)[14] |  |  | severe[7,14] |  |
| *NRXN1* | IS | n=1 w/ ASD (confirmed retrospectively by DSM-IV criteria), IS[15] | 5m[15] | IS[15],GTCS[15] | -lack of eye contact[15]  -lack of social relatedness[15]  -odd way of communication[15]  -repetitive body-rocking[15] |  |  |  |  |
| *PCDH19* | predominantly focal/multifocal epilepsy | n=6 w/ “autistic features”, EFMR or DS [16]  n=3 w/ “autistic features”, EFMR[17]  n=1 w/ “Asperger syndrome”, EFMR [18]  n=1 w/ “autistic features”, EFMR [19]  n=3 w/ “autistic features”, DS (n=3) [20] | 4m-18m[16–20] | AbS[17,20], AS[18,19], ClS[16–18], EMAbs[20], FeS[16–18,20], FS[16,17,20], FSIC[16], GTCS[17,19], HS[16,18], MS[16,20], SE[16,20], TCS[16,20], TS[16,19] | -absent social play[17]  -perseveration[17]  -SIB[17]  -bruxism[17]  -stereotypies (hand flapping, spinning, hand mouthing)[17] | 43% (3/7 DS w/ “autistic features”) [20]  40% (6/15 females w/ “autistic features”)  [16] |  | mild-severe[16,17,19,20] | -ADHD features[16,17]  -aggression[16,17,19]  -anxiety[17]  -obsessions[17,18] |
| *SCN1A* | DS, EIMFS, EMAS, IS, LGS | [10] n=1 w/ “ASD”, LGS  n=11 w/ ASD (based on DSM-IV criteria), DS[21]  n=1 w/ ASD (based on diagnosis by developmental specialists), DS [22] | 14d-35m[10,21,22] | AbS[21], AS[10], CS[10], FeS[21,23], FS[21], FSIC[22], GTCS[21], GTS[10], MS[21,22] |  | 73.3% (11/15 w/ ASD based on DSM-IV criteria)[21] | yes[22] | mild-severe[21] |  |
| *SCN2A* | EIEE, EIMFS, EOEE, IS, LGS | n=1 w/ “autistic behavior”, intractable epilepsy[24]  n=1 w/ “autism”, epileptic encephalopathy [25] | 1y7m-3y[24,25] | AS[24,25], CS[24], other[25] |  |  | yes[24] | severe[24] |  |
| *SCN8A* | EIMFS, EOEE, IS, LGS | n=1 w/ “autism”, epileptic encephalopathy [26] | 6m[26] | GS[26], spasms[26], SUDEP[26] | -obsessive behaviors[26]  -repetitive behaviors[26] |  | yes[26] |  |  |
| *SIK1* | EME, IS | n=3 w/ “autism”, epileptic encephalopathy [27] | 2m-4m[27] | AS[27], GTCS[27], IS[27], MS[27] | -absent speech[27]  -impaired socialization[27]  -repetitive behaviors(bruxism, hand flapping)[27]  -SIB[27] | 50% (3/6 w/ “autism”)[27] |  |  | -aggression[27] |
| *SLC35A2* | EIEE, EOEE, IS | n=1 w/ “ASD”, epileptic encephalopathy [28] | 10m[28] | other[28] | -stereotypies[28] |  |  | severe[28] |  |
| *SLC6A1* | EMAS | n=5 w/ “autistic features”, epileptic encephalopathy [29] | 14m-3y[29] | AAS[29], AbS [29], AS[29], GTCS[29], MAS[29], MS[29], NCSE[29] | -stereotypies[29] | 71% (5/7 w/ “autistic features”)[29] | yes[29] | mild-severe[29] | -aggression[29]  -ADHD[29] |
| *STXBP1* | DS, EIEE, IS, LGS | n=1 w/”PDD”, LGS [10]  n=2 w/ “ASD”, severe ID, IS, MAS (n=1), LGS (n=1)[30]  n=10 w/ “autism or autistic features”, EOEE/WS[31]  n=1 w/ “autistic features”, WS[32] | 1d-22m[10,30–32] | AAS[31],AS[31],CS[31],FS[30–32], FSIC[31],FSSG[31],MAS[30], NCSE[31],spasms[31,32], TCS[31],TS[10,31], GS[33] | -recurrent hand flapping[33]  -stereotyped hand-washing[32] | 28.6% (10/35 w/ “autism or autistic features”)[31] | yes[30] | moderate-profound[30–32] | -agitation[31]  -hyperactivity[31]  -mood lability[31] |

For each gene, classification by epileptic encephalopathy syndromes is based on [34].

Abbreviations

- AAS = atypical absence seizures
- AbS = absence seizures
- ADHD = attention deficit hyperactivity disorder
- AS = atonic seizures
- ASD = autism spectrum disorder
- BNS = benign neonatal seizures
- ClS = seizure clusters
- CS = clonic seizures
- CSWSS = continuous spike-and-wave discharges during slow sleep
- DS = Dravet syndrome
- DSM = Diagnostic and Statistical Manual of Mental Disorders
- EAS = epilepsy-aphasia spectrum
- EIEE = early infantile epileptic encephalopathy
- EIMFS = epilepsy of infancy with migrating focal seizures
- EMAbs = eyelid myoclonia with absences
- EMAS = epilepsy with myoclonic–atonic seizures
- EME = early myoclonic encephalopathy
- EOEE = early-onset epileptic encephalopathy
- FeS = febrile seizures
- FS = focal seizures
- FSIC = CPS = complex partial seizures / focal seizures with impairment of consciousness
- FSSG = focal seizures with secondary generalization
- GeS = gelastic seizures
- GS = generalized seizures
- GTS = generalized tonic seizures
- GTCS = generalized tonic clonic seizures
- HS = hemiclonic seizures
- HyS = hypermotor seizures
- ID = intellectual disability
- IS = infantile spasms
- LGS = Lennox-Gastaut syndrome
- m = months
- MAS = myoclonic-atonic seizures
- MS = myoclonic seizures
- NCSE = non-convulsive status epilepticus
- NFS = nocturnal frontal seizures
- Predominantly focal/multifocal epilepsy
- SE = status epilepticus
- SIB = self-injurious behavior
- SUDEP = sudden unexplained death in epilepsy
- TCS = tonic clonic seizures
- TS = tonic seizures
- TAbS = tonic absence seizuresWS = West syndrome
- y = years

## References

1. Turner G, Partington M, Kerr B, Mangelsdorf M, Gecz J. Variable expression of mental retardation, autism, seizures, and dystonic hand movements in two families with an identical ARX gene mutation. Am. J. Med. Genet. 2002;112:405–11.

2. Archer HL, Evans J, Edwards S, Colley J, Newbury-Ecob R, O’Callaghan F, et al. CDKL5 mutations cause infantile spasms, early onset seizures, and severe mental retardation in female patients. J. Med. Genet. 2006;43:729–34.

3. Zhao Y, Zhang X, Bao X, Zhang Q, Zhang J, Cao G, et al. Clinical features and gene mutational spectrum of CDKL5-related diseases in a cohort of Chinese patients. BMC Med. Genet. 2014;15:24.

4. Maortua H, Martínez-Bouzas C, Calvo M-T, Domingo M-R, Ramos F, García-Ribes A, et al. CDKL5 gene status in female patients with epilepsy and Rett-like features: two new mutations in the catalytic domain. BMC Med. Genet. 2012;13:68.

5. Russo S, Marchi M, Cogliati F, Bonati MT, Pintaudi M, Veneselli E, et al. Novel mutations in the CDKL5 gene, predicted effects and associated phenotypes. Neurogenetics. 2009;10:241–50.

6. Suls A, Jaehn JA, Kecskés A, Weber Y, Weckhuysen S, Craiu DC, et al. De novo loss-of-function mutations in CHD2 cause a fever-sensitive myoclonic epileptic encephalopathy sharing features with Dravet syndrome. Am. J. Hum. Genet. 2013;93:967–75.

7. Carvill GL, Heavin SB, Yendle SC, McMahon JM, O’Roak BJ, Cook J, et al. Targeted resequencing in epileptic encephalopathies identifies de novo mutations in CHD2 and SYNGAP1. Nat. Genet. 2013;45:825–30.

8. Lemke JR, Geider K, Helbig KL, Heyne HO, Schütz H, Hentschel J, et al. Delineating the GRIN1 phenotypic spectrum: A distinct genetic NMDA receptor encephalopathy. Neurology. 2016;86:2171–8.

9. Lesca G, Rudolf G, Bruneau N, Lozovaya N, Labalme A, Boutry-Kryza N, et al. GRIN2A mutations in acquired epileptic aphasia and related childhood focal epilepsies and encephalopathies with speech and language dysfunction. Nat. Genet. 2013;45:1061–6.

10. Epi4K Consortium, Epilepsy Phenome/Genome Project, Allen AS, Berkovic SF, Cossette P, Delanty N, et al. De novo mutations in epileptic encephalopathies. Nature. 2013;501:217–21.

11. Nava C, Dalle C, Rastetter A, Striano P, de Kovel CGF, Nabbout R, et al. De novo mutations in HCN1 cause early infantile epileptic encephalopathy. Nat. Genet. 2014;46:640–5.

12. Gandomi SK, Farwell Gonzalez KD, Parra M, Shahmirzadi L, Mancuso J, Pichurin P, et al. Diagnostic exome sequencing identifies two novel IQSEC2 mutations associated with X-linked intellectual disability with seizures: implications for genetic counseling and clinical diagnosis. J Genet Couns. 2014;23:289–98.

13. Milh M, Boutry-Kryza N, Sutera-Sardo J, Mignot C, Auvin S, Lacoste C, et al. Similar early characteristics but variable neurological outcome of patients with a de novo mutation of KCNQ2. Orphanet J Rare Dis. 2013;8:80.

14. Paciorkowski AR, Traylor RN, Rosenfeld JA, Hoover JM, Harris CJ, Winter S, et al. MEF2C Haploinsufficiency features consistent hyperkinesis, variable epilepsy, and has a role in dorsal and ventral neuronal developmental pathways. Neurogenetics. 2013;14:99.

15. Duong L, Klitten LL, Møller RS, Ingason A, Jakobsen KD, Skjødt C, et al. Mutations in NRXN1 in a family multiply affected with brain disorders: NRXN1 mutations and brain disorders. Am. J. Med. Genet. B Neuropsychiatr. Genet. 2012;159B:354–8.

16. van Harssel JJT, Weckhuysen S, van Kempen MJA, Hardies K, Verbeek NE, de Kovel CGF, et al. Clinical and genetic aspects of PCDH19-related epilepsy syndromes and the possible role of PCDH19 mutations in males with autism spectrum disorders. Neurogenetics. 2013;14:23–34.

17. Jamal SM, Basran RK, Newton S, Wang Z, Milunsky JM. Novel de novo PCDH19 mutations in three unrelated females with epilepsy female restricted mental retardation syndrome. Am. J. Med. Genet. A. 2010;152A:2475–81.

18. Hynes K, Tarpey P, Dibbens LM, Bayly MA, Berkovic SF, Smith R, et al. Epilepsy and mental retardation limited to females with PCDH19 mutations can present de novo or in single generation families. J. Med. Genet. 2010;47:211–6.

19. Dimova PS, Kirov A, Todorova A, Todorov T, Mitev V. A novel PCDH19 mutation inherited from an unaffected mother. Pediatr. Neurol. 2012;46:397–400.

20. Marini C, Mei D, Parmeggiani L, Norci V, Calado E, Ferrari A, et al. Protocadherin 19 mutations in girls with infantile-onset epilepsy. Neurology. 2010;75:646–53.

21. Kwong AK-Y, Fung C-W, Chan S-Y, Wong VC-N. Identification of SCN1A and PCDH19 mutations in Chinese children with Dravet syndrome. PLoS ONE. 2012;7:e41802.

22. Frosk P, Mhanni AA, Rafay MF. SCN1A mutation associated with intractable myoclonic epilepsy and migraine headache. J. Child Neurol. 2013;28:389–91.

23. Tan EH, Yusoff AAM, Abdullah JM, Razak SA. Generalized epilepsy with febrile seizure plus (GEFS+) spectrum: Novel de novo mutation of SCN1A detected in a Malaysian patient. J Pediatr Neurosci. 2012;7:123–5.

24. Kamiya K, Kaneda M, Sugawara T, Mazaki E, Okamura N, Montal M, et al. A nonsense mutation of the sodium channel gene SCN2A in a patient with intractable epilepsy and mental decline. J. Neurosci. 2004;24:2690–8.

25. Horvath GA, Demos M, Shyr C, Matthews A, Zhang L, Race S, et al. Secondary neurotransmitter deficiencies in epilepsy caused by voltage-gated sodium channelopathies: A potential treatment target? Mol. Genet. Metab. 2016;117:42–8.

26. Veeramah KR, O’Brien JE, Meisler MH, Cheng X, Dib-Hajj SD, Waxman SG, et al. De novo pathogenic SCN8A mutation identified by whole-genome sequencing of a family quartet affected by infantile epileptic encephalopathy and SUDEP. Am. J. Hum. Genet. 2012;90:502–10.

27. Hansen J, Snow C, Tuttle E, Ghoneim DH, Yang C-S, Spencer A, et al. De novo mutations in SIK1 cause a spectrum of developmental epilepsies. Am. J. Hum. Genet. 2015;96:682–90.

28. Lopes F, Barbosa M, Ameur A, Soares G, de Sá J, Dias AI, et al. Identification of novel genetic causes of Rett syndrome-like phenotypes. J. Med. Genet. 2016;

29. Carvill GL, McMahon JM, Schneider A, Zemel M, Myers CT, Saykally J, et al. Mutations in the GABA Transporter SLC6A1 Cause Epilepsy with Myoclonic-Atonic Seizures. Am. J. Hum. Genet. 2015;96:808–15.

30. Campbell IM, Yatsenko SA, Hixson P, Reimschisel T, Thomas M, Wilson W, et al. Novel 9q34.11 gene deletions encompassing combinations of four Mendelian disease genes: STXBP1, SPTAN1, ENG, and TOR1A. Genet. Med. 2012;14:868–76.

31. Stamberger H, Nikanorova M, Willemsen MH, Accorsi P, Angriman M, Baier H, et al. STXBP1 encephalopathy: A neurodevelopmental disorder including epilepsy. Neurology. 2016;86:954–62.

32. Romaniello R, Saettini F, Panzeri E, Arrigoni F, Bassi MT, Borgatti R. A de-novo STXBP1 gene mutation in a patient showing the Rett syndrome phenotype. Neuroreport. 2015;26:254–7.

33. Deciphering Developmental Disorders Study. Large-scale discovery of novel genetic causes of developmental disorders. Nature. 2015;519:223–8.

34. McTague A, Howell KB, Cross JH, Kurian MA, Scheffer IE. The genetic landscape of the epileptic encephalopathies of infancy and childhood. Lancet Neurol. 2016;15:304–16.
