# Supplementary material for: Autism spectrum disorder and epileptic encephalopathy: common causes, many questions
Source: J Neurodev Disord. 2017 Jun 23;9:23. doi: 10.1186/s11689-017-9202-0 (PMC5481888; doi:10.1186/s11689-017-9202-0)
Supplement: Supplementary file 2 — Evidence linking epileptic encephalopathy genes to ASD. (DOCX 218 kb) [file 11689_2017_9202_MOESM2_ESM.docx]

## Table S2. Evidence linking epileptic encephalopathy genes to ASD.

| **Gene** | **Variant Discovery in ASD Cohorts** | | | **Variant Discovery in Other Cohorts** | | **Phenotype Studies of Patients with Variants Affecting Gene of Interest** | |
| --- | --- | --- | --- | --- | --- | --- | --- |
|  | Whole-exome or whole-genome sequencing (unless otherwise noted) of ASD cohorts revealing intragenic variants in the gene of interest | SNP association studies of ASD cohorts revealing SNPs in the gene of interest | Chromosomal analysis of ASD cohorts revealing CNVs disrupting only the gene of interest or regulatory elements of the gene | Whole-exome or whole-genome sequencing (unless otherwise noted) of ID or epilepsy cohorts revealing intragenic variants in the gene of interest in at least one patient with ASD or features of ASD | Chromosomal analysis of ID or epilepsy cohorts revealing CNVs disrupting only the gene of interest or regulatory elements of the gene | Case reports/series of patients with intragenic variants in the gene of interest, with at least one of the patients demonstrating ASD or features of ASD | Case reports/series of patients with a CNV or other chromosomal anomaly disrupting only the gene of interest, with at least one of the patients demonstrating ASD or features of ASD |
| *ARX* | -Well-defined ASD cohort [1,2] |  |  |  |  | -*ARX* variants cohort (n=50 affected males from 9 families): n=4 from 2 families w/ “autism”, ± epilepsy, ID [3]  -*ARX* variants cohort (multiple affected individuals from 2 families): n=3 from 2 families w/ “autism” or “autistic behavior”, epilepsy (n=2 including n=1 w/ IS), severe ID [4]  -*ARX* variants cohort (n=4 affected individuals from 3 families): n=1 w/ well-defined ASD, seizures, GDD [5] |  |
| *CACNA1A* |  |  |  |  |  | -*CACNA1A* variants, “spectrum of cognitive impairment” cohort (n=13 from 3 families w/ an intragenic variant plus n=3 from 1 family w/ a CNV): n=2 from 1 family w/ “ASD”, ± seizures (n=1), GDD [6] | -CNV (maternally inherited 19p13.13 microdeletion affecting *CACNA1A*) case report: n=1 from 1 family w/ well-defined ASD, seizures, mild ID [6] |
| *CDKL5* | -Well-defined ASD cohort [2,7] |  |  |  | -Epilepsy ± neurodevelopmental disorders cohort [8] | -*CDKL5* variants cohort (from n=73 who underwent *CDKL5* sequencing, n=7 w/ pathogenic *CDKL5* variants): n=1 w/ WS, “autism”, severe ID [9]-*CDKL5* variants cohort (from n=102 w/ EOEE and/or *MECP2* mutation-negative RTT who underwent *CDKL5* sequencing, n=10 w/ *CDKL5* variants): n=10 w/ “autistic features”, epileptic encephalopathy [10]*-CDKL5* variants case series (from n=60 females w/ epilepsy, negative *MECP2* sequencing who underwent *CDKL5* sequencing and MLPA analysis, n=2 w/ variants in catalytic domain): n=2 w/ epileptic encephalopathy, regression, ID, “autistic characteristics”, ± RTT clinical diagnosis (n=1) [11]-*CDKL5* variant case report: from a family w/ members affected by neurodevelopmental diagnoses, n=1 w/ “autism”, no epilepsy (her identical twin w/ severe ID, epilepsy, and clinical diagnosis of atypical RTT) [12] *-CDKL5* variants cohort (from n=92 w/ clinical diagnosis of RTT, Angelman syndrome features, or ASD who underwent *CDKL5* sequencing, n=7 w/ *CDKL5* variants): n=1 w/ intractable epilepsy, severe ID, “autistic” features [13] | -*CDKL5* variants case series (from n=7 unrelated families w/ duplications affecting *CDKL5*): n=5 w/ “ASD”, no epilepsy [14] |
| *CHD2* | -Well-defined ASD cohort [1,15–17] |  |  | -Epileptic encephalopathy cohort undergoing targeted parallel sequencing of genes associated with epileptic encephalopathy [18] |  | -*CHD2* variant case report: n=1 w/ “ASD”, ID, myoclonic epileptic encephalopathy [19] |  |
| *ERBB4* |  |  | -Well-defined ASD cohort [20,21] |  |  |  |  |
| *FLNA* |  |  | -Well-defined ASD [22] |  |  |  |  |
| *FOXG1* | -Well-defined ASD cohort [15]  -Well-defined ASD cohort, undergoing targeted NGS panel [23] |  |  |  |  | *FOXG1* variants cohort (n=11 new or previously characterized subjects, who had socialization characterized, w/ intragenic variants): n=11/11 w/  “poor social interaction” “denoting a syndromic form of autism” ± epilepsy[24] |  |
| *GABRA1* | -Well-defined ASD cohort[1] |  |  |  |  |  |  |
| *GABRB1* | -Well-defined ASD cohort [15] | -Well-defined ASD cohort [25,26] |  |  |  |  |  |
| *GABRB3* | -Well-defined ASD cohort [15,17,27,28] | -Well-defined ASD cohort [29–32] |  |  |  |  |  |
| *GABRG2* |  | -Well-defined ASD cohort [33] |  |  |  |  |  |
| *GRIN1* |  |  |  |  |  | -*GRIN1* variants cohort (n=23 w/ heterozygous variants plus n=5 from 2 families w/ homozygous variants): n=8/23 w/ “ASD” or “ASD-like features”, ± epilepsy or epileptic encephalopathy, severe ID [34] |  |
| *GRIN2A* | -Well-defined ASD (brain specimens) [35] | -Well-defined ASD cohort [36] | -Well-defined ASD cohort [21] |  |  | -*GRIN2A* variants, epileptic aphasia spectrum cohort (n=36 comprising n=29 patients from 9 families plus n=7 non-related individuals): n=2 patients (n=1 of the non-related individuals and n=1 from a family) w/ “autistic features”, CSWSS, ± GDD w/out regression (n=1), ± normal development followed by language predominant global regression (n=1) [37] |  |
| *GRIN2B* | -Well-defined ASD cohort [15,17,38,39]  -Well-defined ASD cohort, undergoing targeted NGS panel [40]  -Well-defined ASD cohort, focusing on NMDA receptor subunit genes [41] | -Well-defined ASD cohort [42,43] | -Well-defined ASD [44] | -Epileptic encephalopathy cohort [45] |  |  |  |
| *HCN1* |  |  |  |  |  | -*HCN1* variants, EIEE cohort (n=6): n=4 w/ “autistic features”, multiple seizure types, moderate-severe ID [46] |  |
| *IQSEC2* |  |  |  |  |  | -*IQSEC2* variants cohort (n=2): n=2 w/ “autistic-like features”, intractable epilepsy, DD [47]  -*IQSEC2* variants, XLID cohort (n=4 families): n=2 families affected by “autistic features”, moderate to severe ID [48]  -*IQSEC2* variant, XLID case report (n=14 individuals from 1 family): n=2 individuals w/ “PDD”, “ASD” (or suspicion of), no seizures, moderate to severe ID [49]  -*IQSEC2* variants cohort (n=3): n=3 w/ “autism-like behaviour”, ± epilepsy(n=2), ± regression (n=1), severe ID [50] |  |
| *KCNQ2* | -Well-defined ASD cohort [16,51] |  |  |  |  | -*KCNQ2* variants, EOEE cohort (n=16): n=1 w/ “autistic features”, multiple seizure types, DD [52] |  |
| *KCNQ3* | -Well-defined ASD cohort [15,53] |  |  |  |  | -*KCNQ3* variants cohort (n=3): n=3 w/ well-defined ASD, no seizures, low average IQ to mild ID [54] | -CNV (*de novo* t(3;8) (q21;q24) translocation truncating *KCNQ3*) case report: n=1 w/ well-defined ASD, possible h/o benign neonatal convulsions, no epilepsy diagnosis, low average IQ [54] |
| *MEF2C* | -Well-defined ASD cohort [55] |  |  | -Epileptic encephalopathy cohort undergoing targeted parallel sequencing of genes associated with epileptic encephalopathy[18]  -Severe ID cohort undergoing  *MEF2C* sequencing [56] |  | *MEF2C* variants cohort (  from n=15 w/ *MEF2C* deletions or intragenic variants,  n=1 w/ intragenic *MEF2C* variant): n=1 w/ stereotypies (hand wringing) consistent with an “autism plus” phenotype, myoclonic atonic epilepsy [57] | -CNV (5q14.3 microdeletion affecting *MEF2C*) case report: n=1 w/ “ASD”, epilepsy, severe ID [58]  *MEF2C* variants cohort (  from n=15 w/ *MEF2C* deletions or intragenic variants, n=1 w/ 5q14.3 microdeletion affecting only *MEF2C)*: n=1 w/ stereotypies (hand batting, head shaking, bruxism) consistent with an “autism plus” phenotype, no epilepsy [57] |
| *MTOR* | -Well-defined ASD cohort [1] |  |  | -Focal cortical dysplasia, hemimegalencephaly, megalencephaly cohort [59] |  | -*MTOR* variant case report: n=2 brothers w/ “ASD”, mild-moderate ID, megalencephaly [60] |  |
| *NRXN1* | -Well-defined ASD cohort, focusing on *NRXN1-3* mutation scanning [61]  -Well-defined ASD cohort, focusing on *NRXN1* sequencing [62–64]  -Well-defined ASD and ID cohort, focusing on *NRXN1* sequencing [65]  -Well-defined ASD cohort [15,51]  -Well-defined ASD cohort, undergoing targeted NGS panel [66]  -Well-defined ASD cohort [17,27] | -Well-defined ASD cohort [67] | -Well-defined ASD cohort [20,21,68–70] | -Pitt-Hopkins syndrome-like phenotype undergoing sequencing of *CNTNAP2* and *NRXN1* [71] | -ID cohort [72] | -*NRXN1* variant (compound heterozygous variant with inherited promoter/intronic deletion plus inherited point mutation) case report: n=1 (among several affected family members) w/ well-defined ASD, ID, IS [73] | -CNV (biallelic 2p16.3 deletions disrupting *NRXN1*) case report: n=2 (fraternal twins) w/ “autism”/”autistic disorder”, severe ID, epilepsy (n=1) [74]  -CNVs (exon deletions disrupting *NRXN1*) cohort (n=23 w/ detailed clinical information): n=13 w/ well-defined ASD, ± ID, ± seizures [75]  -CNVs (exon deletions disrupting *NRXN1*) cohort (n=12): n=4 w/ well-defined ASD [76]  -CNVs (deletions disrupting *NRXN1*) cohort (n=44 w/ clinical data): n=14 w/ “ASD” [77] |
| *PCDH19* | -Well-defined ASD cohort, undergoing sequencing of X-linked synaptic genes [78] |  |  | -EFMR cohort undergoing sequencing of select X-linked genes[79] |  | -*PCDH19* variants, females, epilepsy cohort (n=13 w/ positive *PCDH19* screening among n=117 females w/ FeS and wide spectrum of epilepsy phenotypes): n=6 w/ “autistic features”, DS (n=3), focal epilepsy (n=3), mild-severe ID [80]  -*PCDH19* variants, early onset epilepsy cohort (n=11): n=5 w/ well-defined ASD, multiple seizure types, moderate-severe ID [81]  -*PCDH19* variants, early onset epilepsy cohort (n=18): n=13 w/ “autistic traits”, multiple seizure types, mild-severe ID [82] -*PCDH19* variants cohort (n=15): n=6 w/ “autistic features”, EFMR or DS, moderate ID [83] -*PCDH19* variants, epilepsy cohort (n=35): n=10 w/ “autistic features”, focal seizures [84]-*PCDH19* variant case report: n=1 w/ “autistic features”, EFMR, moderate ID [85]  -*PCDH19* variants, EFMR cohort (n=3): n=3 w/ “autistic features”, EFMR, mild-moderate ID [86]  -*PCDH19* variants, EFMR cohort (n=3 including 2 sisters from 1 family): n=1 w/ “Asperger syndrome”, EFMR, average IQ [87]-*PCDH19* variant case report (from n=100 w/ severe epilepsy who underwent *SCN1A* sequencing and if negative then *PCDH19* sequencing): n=1 w/ well-defined ASD, multiple seizure types, mild ID [88] |  |
| *PLCB1* |  |  | -Well-defined ASD cohort [21,68,89] |  |  |  |  |
| *PTEN* | -Well-defined ASD and macrocephaly cohort, focusing on *PTEN* sequencing [90,91]  Well-defined ASD cohort, focusing on *PTEN* sequencing [92]  -Well-defined ASD (brain specimens) [35]  -Well-defined ASD cohort, undergoing targeted NGS panel [40] -Well-defined ASD cohort [15,17,70] |  |  |  |  | -*PTEN* variant case report: n=1 w/ well-defined ASD, ID [93]  -*PTEN* variants cohort (among n=93 w/ ASD and/or ID who underwent *PTEN* sequencing, n=4 w/ *PTEN* variants): n=2 w/ well-defined ASD, macrocephaly [94]  -*PTEN* variants cohort (n=23 w/ *PTEN* variants): n=8 w/ “autistic features”/”autistic spectrum”/”PDD” [95]  -*PTEN* variants cohort: n=17 w/ well-defined ASD [96] |  |
| *SCN1A* | -Well-defined ASD cohort, focusing on *SCN1A*, *SCN2A*, *SCN3A* sequencing [97]  -Well-defined ASD cohort [38,53,98]  -Well-defined ASD cohort, undergoing targeted NGS panel [23,40]  -Well-defined ASD cohort (brain specimens) [35] |  |  | -Epileptic encephalopathy cohort [45] |  | -*SCN1A* variants case series (from n=100 w/ DS who underwent *SCN1A* sequencing and if negative then *PCDH19* sequencing, n=15 w/ *SCN1A* variants): n=11/15 w/ well-defined ASD, mild-severe ID, multiple seizure types [88]-*SCN1A* variant case report: n=1 w/ “autism features”, regression, FeS, GTCS, ID [99]-*SCN1A* variant case report: n=1 w/ well-defined ASD, DS, regression, GDD [100]  -*SCN1A* variant case report: from a family, n=1 w/ GTCS, well-defined ASD (Asperger disorder) [101] |  |
| *SCN8A* |  |  |  |  |  | -*SCN8A* variant case report: n=1 w/ “autism”, epileptic encephalopathy (culminating in SUDEP), ID [102]  -*SCN8A* variants case series (n=3): n=1 w/ “ASD”, no seizures, mild ID [103] |  |
| *SCN2A* | -Well-defined ASD cohort [1,7,15,27,28,51,70,104,105]  -Well-defined ASD cohort focusing on *SCN1A*, *SCN2A*, *SCN3A* sequencing [97]  -Well-defined ASD cohort (brain specimens) [35] |  |  | -Severe developmental disorders cohort [106]  -Severe non-syndromic ID cohort [107] |  | -*SCN2A* variant case report: n=1 w/ well-defined ASD [108]  -*SCN2A* variants, intractable epilepsy cohort (n=2): n=1 w/ “autism”, epileptic encephalopathy[109]  -*SCN2A* variant case report: n=1 w/ “autistic behavior”, intractable epilepsy, severe ID, severe cognitive decline [110] |  |
| *SETBP1* | -Well-defined ASD cohort [1,15,98] |  |  | -Severe non-syndromic ID cohort [107]  -DD or “ASD” cohort[111] |  |  |  |
| *SIK1* | -Well-defined ASD cohort [1] |  |  |  |  | -*SIK1* variants, epileptic encephalopathy (EME, OS, IS) cohort (n=6): n=3 w/“autism”, intractable epilepsy, IS, ID [112] |  |
| *SLC12A5* | -Well-defined ASD, focusing on *SLC12A5* sequencing [113] |  |  |  |  |  |  |
| *SLC35A2* |  |  |  |  |  | -*SLC35A2* variant case report (among other disorders w/ features of RTT): n=1 w/ “autism features”, epileptic encephalopathy, severe ID [114] |  |
| *SLC6A1* | -Well-defined ASD cohort [1,15,17,28,105] |  |  |  |  | -*SLC6A1* variants, MAS cohort (n=7): n=5 w/ “autistic features”, multiple seizure types, mild to severe ID, ± regression (n=2) [115] |  |
| *STXBP1* | -Well-defined ASD cohort [55,104] |  |  | -Epileptic encephalopathy cohort [45]  -Severe non-syndromic ID cohort [107]  -Severe developmental disorders cohort [106] |  | -*STXBP1* variant case report: n=1 w/ “autistic features”, WS, profound ID [116]  -*STXBP1* variants cohort (n=45): n=14 w/ “autism or autistic features” [117] | -CNVs (9q34.11 deletions disrupting *STXBP1*) cohort: n=2 w/ “ASD”, severe ID, IS, MAS (n=1), LGS (n=1)[118] |
| *TCF4* | -Well-defined ASD cohort [15] |  |  | -Unexplained ID cohort [119] |  | -*TCF4* variants cohort (n=10): n=10 w/ features of well-defined ASD, profound ID, ± epilepsy (n=1) [120] |  |

We use the phrase “well-defined ASD” to indicate that the patients in the study received a diagnosis of ASD through standardized methodology, such as DSM-IV or DSM-5 criteria, ADOS testing, and/or ADI-R testing. Otherwise, we use quotation marks around specific words/phrases that pertain to how the study authors described the presence of ASD

## Abbreviations

- ADI-R = Autism Diagnostic Interview-Revised
- ADOS = Autism Diagnostic Observation Schedule
- ASD = autism spectrum disorderCNV = copy number variant
- CSWSS = continuous spike-wave discharges in slow wave sleep syndrome
- DD = developmental delay
- DS = Dravet syndrome
- DSM = Diagnostic and Statistical Manual of Mental Disorders
- EFMR = epilepsy-intellectual disability in femalesEIEE = early infantile epileptic encephalopathy
- EME = early myoclonic encephalopathy
- EOEE = early onset epileptic encephalopathy
- FeS = febrile seizures
- GDD = global developmental delay
- GTCS = generalized tonic clonic seizures
- h/o = history of
- ID = intellectual disability
- IS = infantile spasms
- LGS = Lennox-Gastaut syndrome
- MAS = myoclonic-atonic seizures
- MLPA = multiplex ligation-dependent probe amplification
- NGS = next-generation sequencing
- OS = Ohtahara syndrome
- RTT = Rett syndrome
- SNP = single-nucleotide polymorphism
- SUDEP = sudden unexpected death in epilepsy
- w/ = with
- WS = West syndrome
- XLID = X-linked ID

## References

1. Iossifov I, O’Roak BJ, Sanders SJ, Ronemus M, Krumm N, Levy D, et al. The contribution of de novo coding mutations to autism spectrum disorder. Nature. 2014;515:216–21.

2. Schaaf CP, Sabo A, Sakai Y, Crosby J, Muzny D, Hawes A, et al. Oligogenic heterozygosity in individuals with high-functioning autism spectrum disorders. Hum. Mol. Genet. 2011;20:3366–75.

3. Strømme P, Mangelsdorf ME, Scheffer IE, Gécz J. Infantile spasms, dystonia, and other X-linked phenotypes caused by mutations in Aristaless related homeobox gene, ARX. Brain Dev. 2002;24:266–8.

4. Turner G, Partington M, Kerr B, Mangelsdorf M, Gecz J. Variable expression of mental retardation, autism, seizures, and dystonic hand movements in two families with an identical ARX gene mutation. Am. J. Med. Genet. 2002;112:405–11.

5. Wallerstein R, Sugalski R, Cohn L, Jawetz R, Friez M. Expansion of the ARX spectrum. Clin Neurol Neurosurg. 2008;110:631–4.

6. Damaj L, Lupien-Meilleur A, Lortie A, Riou É, Ospina LH, Gagnon L, et al. CACNA1A haploinsufficiency causes cognitive impairment, autism and epileptic encephalopathy with mild cerebellar symptoms. Eur. J. Hum. Genet. 2015;23:1505–12.

7. Codina-Solà M, Rodríguez-Santiago B, Homs A, Santoyo J, Rigau M, Aznar-Laín G, et al. Integrated analysis of whole-exome sequencing and transcriptome profiling in males with autism spectrum disorders. Mol Autism. 2015;6:21.

8. Bartnik M, Szczepanik E, Derwińska K, Wiśniowiecka-Kowalnik B, Gambin T, Sykulski M, et al. Application of array comparative genomic hybridization in 102 patients with epilepsy and additional neurodevelopmental disorders. Am. J. Med. Genet. B Neuropsychiatr. Genet. 2012;159B:760–71.

9. Archer HL, Evans J, Edwards S, Colley J, Newbury-Ecob R, O’Callaghan F, et al. CDKL5 mutations cause infantile spasms, early onset seizures, and severe mental retardation in female patients. J. Med. Genet. 2006;43:729–34.

10. Zhao Y, Zhang X, Bao X, Zhang Q, Zhang J, Cao G, et al. Clinical features and gene mutational spectrum of CDKL5-related diseases in a cohort of Chinese patients. BMC Med. Genet. 2014;15:24.

11. Maortua H, Martínez-Bouzas C, Calvo M-T, Domingo M-R, Ramos F, García-Ribes A, et al. CDKL5 gene status in female patients with epilepsy and Rett-like features: two new mutations in the catalytic domain. BMC Med. Genet. 2012;13:68.

12. Weaving LS, Christodoulou J, Williamson SL, Friend KL, McKenzie OLD, Archer H, et al. Mutations of CDKL5 cause a severe neurodevelopmental disorder with infantile spasms and mental retardation. Am. J. Hum. Genet. 2004;75:1079–93.

13. Russo S, Marchi M, Cogliati F, Bonati MT, Pintaudi M, Veneselli E, et al. Novel mutations in the CDKL5 gene, predicted effects and associated phenotypes. Neurogenetics. 2009;10:241–50.

14. Szafranski P, Golla S, Jin W, Fang P, Hixson P, Matalon R, et al. Neurodevelopmental and neurobehavioral characteristics in males and females with CDKL5 duplications. Eur. J. Hum. Genet. 2015;23:915–21.

15. De Rubeis S, He X, Goldberg AP, Poultney CS, Samocha K, Cicek AE, et al. Synaptic, transcriptional and chromatin genes disrupted in autism. Nature. 2014;515:209–15.

16. Dong S, Walker MF, Carriero NJ, DiCola M, Willsey AJ, Ye AY, et al. De novo insertions and deletions of predominantly paternal origin are associated with autism spectrum disorder. Cell Rep. 2014;9:16–23.

17. Iossifov I, Levy D, Allen J, Ye K, Ronemus M, Lee Y-H, et al. Low load for disruptive mutations in autism genes and their biased transmission. Proc. Natl. Acad. Sci. U.S.A. 2015;112:E5600-5607.

18. Carvill GL, Heavin SB, Yendle SC, McMahon JM, O’Roak BJ, Cook J, et al. Targeted resequencing in epileptic encephalopathies identifies de novo mutations in CHD2 and SYNGAP1. Nat. Genet. 2013;45:825–30.

19. Suls A, Jaehn JA, Kecskés A, Weber Y, Weckhuysen S, Craiu DC, et al. De novo loss-of-function mutations in CHD2 cause a fever-sensitive myoclonic epileptic encephalopathy sharing features with Dravet syndrome. Am. J. Hum. Genet. 2013;93:967–75.

20. Pinto D, Pagnamenta AT, Klei L, Anney R, Merico D, Regan R, et al. Functional impact of global rare copy number variation in autism spectrum disorders. Nature. 2010;466:368–72.

21. Girirajan S, Dennis MY, Baker C, Malig M, Coe BP, Campbell CD, et al. Refinement and discovery of new hotspots of copy-number variation associated with autism spectrum disorder. Am. J. Hum. Genet. 2013;92:221–37.

22. Sakai Y, Shaw CA, Dawson BC, Dugas DV, Al-Mohtaseb Z, Hill DE, et al. Protein interactome reveals converging molecular pathways among autism disorders. Sci Transl Med. 2011;3:86ra49.

23. Alvarez-Mora MI, Calvo Escalona R, Puig Navarro O, Madrigal I, Quintela I, Amigo J, et al. Comprehensive molecular testing in patients with high functioning autism spectrum disorder. Mutat. Res. 2016;784–785:46–52.

24. Kortüm F, Das S, Flindt M, Morris-Rosendahl DJ, Stefanova I, Goldstein A, et al. The core FOXG1 syndrome phenotype consists of postnatal microcephaly, severe mental retardation, absent language, dyskinesia, and corpus callosum hypogenesis. J. Med. Genet. 2011;48:396–406.

25. Collins AL, Ma D, Whitehead PL, Martin ER, Wright HH, Abramson RK, et al. Investigation of autism and GABA receptor subunit genes in multiple ethnic groups. Neurogenetics. 2006;7:167–74.

26. Ma DQ, Whitehead PL, Menold MM, Martin ER, Ashley-Koch AE, Mei H, et al. Identification of significant association and gene-gene interaction of GABA receptor subunit genes in autism. Am. J. Hum. Genet. 2005;77:377–88.

27. Iossifov I, Ronemus M, Levy D, Wang Z, Hakker I, Rosenbaum J, et al. De novo gene disruptions in children on the autistic spectrum. Neuron. 2012;74:285–99.

28. Krumm N, Turner TN, Baker C, Vives L, Mohajeri K, Witherspoon K, et al. Excess of rare, inherited truncating mutations in autism. Nat. Genet. 2015;47:582–8.

29. Cook EH, Courchesne RY, Cox NJ, Lord C, Gonen D, Guter SJ, et al. Linkage-disequilibrium mapping of autistic disorder, with 15q11-13 markers. Am. J. Hum. Genet. 1998;62:1077–83.

30. Warrier V, Baron-Cohen S, Chakrabarti B. Genetic variation in GABRB3 is associated with Asperger syndrome and multiple endophenotypes relevant to autism. Mol Autism. 2013;4:48.

31. Buxbaum JD, Silverman JM, Smith CJ, Greenberg DA, Kilifarski M, Reichert J, et al. Association between a GABRB3 polymorphism and autism. Mol. Psychiatry. 2002;7:311–6.

32. Delahanty RJ, Kang JQ, Brune CW, Kistner EO, Courchesne E, Cox NJ, et al. Maternal transmission of a rare GABRB3 signal peptide variant is associated with autism. Mol. Psychiatry. 2011;16:86–96.

33. Sesarini CV, Costa L, Grañana N, Coto MG, Pallia RC, Argibay PF. Association between GABA(A) receptor subunit polymorphisms and autism spectrum disorder (ASD). Psychiatry Res. 2015;229:580–2.

34. Lemke JR, Geider K, Helbig KL, Heyne HO, Schütz H, Hentschel J, et al. Delineating the GRIN1 phenotypic spectrum: A distinct genetic NMDA receptor encephalopathy. Neurology. 2016;86:2171–8.

35. D’Gama AM, Pochareddy S, Li M, Jamuar SS, Reiff RE, Lam A-TN, et al. Targeted DNA Sequencing from Autism Spectrum Disorder Brains Implicates Multiple Genetic Mechanisms. Neuron. 2015;88:910–7.

36. Barnby G, Abbott A, Sykes N, Morris A, Weeks DE, Mott R, et al. Candidate-gene screening and association analysis at the autism-susceptibility locus on chromosome 16p: evidence of association at GRIN2A and ABAT. Am. J. Hum. Genet. 2005;76:950–66.

37. Lesca G, Rudolf G, Bruneau N, Lozovaya N, Labalme A, Boutry-Kryza N, et al. GRIN2A mutations in acquired epileptic aphasia and related childhood focal epilepsies and encephalopathies with speech and language dysfunction. Nat. Genet. 2013;45:1061–6.

38. O’Roak BJ, Deriziotis P, Lee C, Vives L, Schwartz JJ, Girirajan S, et al. Exome sequencing in sporadic autism spectrum disorders identifies severe de novo mutations. Nat Genet. 2011;43:585–9.

39. Kenny EM, Cormican P, Furlong S, Heron E, Kenny G, Fahey C, et al. Excess of rare novel loss-of-function variants in synaptic genes in schizophrenia and autism spectrum disorders. Mol. Psychiatry. 2014;19:872–9.

40. O’Roak BJ, Vives L, Fu W, Egertson JD, Stanaway IB, Phelps IG, et al. Multiplex targeted sequencing identifies recurrently mutated genes in autism spectrum disorders. Science. 2012;338:1619–22.

41. Tarabeux J, Kebir O, Gauthier J, Hamdan FF, Xiong L, Piton A, et al. Rare mutations in N-methyl-D-aspartate glutamate receptors in autism spectrum disorders and schizophrenia. Transl Psychiatry. 2011;1:e55.

42. Pan Y, Chen J, Guo H, Ou J, Peng Y, Liu Q, et al. Association of genetic variants of GRIN2B with autism. Sci Rep. 2015;5:8296.

43. Yoo HJ, Cho IH, Park M, Yang SY, Kim SA. Family based association of GRIN2A and GRIN2B with Korean autism spectrum disorders. Neurosci. Lett. 2012;512:89–93.

44. Talkowski ME, Rosenfeld JA, Blumenthal I, Pillalamarri V, Chiang C, Heilbut A, et al. Sequencing chromosomal abnormalities reveals neurodevelopmental loci that confer risk across diagnostic boundaries. Cell. 2012;149:525–37.

45. Epi4K Consortium, Epilepsy Phenome/Genome Project, Allen AS, Berkovic SF, Cossette P, Delanty N, et al. De novo mutations in epileptic encephalopathies. Nature. 2013;501:217–21.

46. Nava C, Dalle C, Rastetter A, Striano P, de Kovel CGF, Nabbout R, et al. De novo mutations in HCN1 cause early infantile epileptic encephalopathy. Nat. Genet. 2014;46:640–5.

47. Gandomi SK, Farwell Gonzalez KD, Parra M, Shahmirzadi L, Mancuso J, Pichurin P, et al. Diagnostic exome sequencing identifies two novel IQSEC2 mutations associated with X-linked intellectual disability with seizures: implications for genetic counseling and clinical diagnosis. J Genet Couns. 2014;23:289–98.

48. Shoubridge C, Tarpey PS, Abidi F, Ramsden SL, Rujirabanjerd S, Murphy JA, et al. Mutations in the guanine nucleotide exchange factor gene IQSEC2 cause nonsyndromic intellectual disability. Nat. Genet. 2010;42:486–8.

49. Kalscheuer VM, James VM, Himelright ML, Long P, Oegema R, Jensen C, et al. Novel Missense Mutation A789V in IQSEC2 Underlies X-Linked Intellectual Disability in the MRX78 Family. Front Mol Neurosci. 2015;8:85.

50. Tran Mau-Them F, Willems M, Albrecht B, Sanchez E, Puechberty J, Endele S, et al. Expanding the phenotype of IQSEC2 mutations: truncating mutations in severe intellectual disability. Eur. J. Hum. Genet. 2014;22:289–92.

51. Jiang Y, Yuen RKC, Jin X, Wang M, Chen N, Wu X, et al. Detection of clinically relevant genetic variants in autism spectrum disorder by whole-genome sequencing. Am. J. Hum. Genet. 2013;93:249–63.

52. Milh M, Boutry-Kryza N, Sutera-Sardo J, Mignot C, Auvin S, Lacoste C, et al. Similar early characteristics but variable neurological outcome of patients with a de novo mutation of KCNQ2. Orphanet J Rare Dis. 2013;8:80.

53. Toma C, Torrico B, Hervás A, Valdés-Mas R, Tristán-Noguero A, Padillo V, et al. Exome sequencing in multiplex autism families suggests a major role for heterozygous truncating mutations. Mol. Psychiatry. 2014;19:784–90.

54. Gilling M, Rasmussen HB, Calloe K, Sequeira AF, Baretto M, Oliveira G, et al. Dysfunction of the Heteromeric KV7.3/KV7.5 Potassium Channel is Associated with Autism Spectrum Disorders. Front Genet. 2013;4:54.

55. Neale BM, Kou Y, Liu L, Ma’ayan A, Samocha KE, Sabo A, et al. Patterns and rates of exonic de novo mutations in autism spectrum disorders. Nature. 2012;485:242–5.

56. Zweier M, Gregor A, Zweier C, Engels H, Sticht H, Wohlleber E, et al. Mutations in MEF2C from the 5q14.3q15 microdeletion syndrome region are a frequent cause of severe mental retardation and diminish MECP2 and CDKL5 expression. Hum. Mutat. 2010;31:722–33.

57. Paciorkowski AR, Traylor RN, Rosenfeld JA, Hoover JM, Harris CJ, Winter S, et al. MEF2C Haploinsufficiency features consistent hyperkinesis, variable epilepsy, and has a role in dorsal and ventral neuronal developmental pathways. Neurogenetics. 2013;14:99.

58. Novara F, Beri S, Giorda R, Ortibus E, Nageshappa S, Darra F, et al. Refining the phenotype associated with MEF2C haploinsufficiency. Clin. Genet. 2010;78:471–7.

59. Mirzaa GM, Campbell CD, Solovieff N, Goold CP, Jansen LA, Menon S, et al. Association of MTOR Mutations With Developmental Brain Disorders, Including Megalencephaly, Focal Cortical Dysplasia, and Pigmentary Mosaicism. JAMA Neurol. 2016;73:836–45.

60. Mroske C, Rasmussen K, Shinde DN, Huether R, Powis Z, Lu H-M, et al. Germline activating MTOR mutation arising through gonadal mosaicism in two brothers with megalencephaly and neurodevelopmental abnormalities. BMC Med. Genet. 2015;16:102.

61. Feng J, Schroer R, Yan J, Song W, Yang C, Bockholt A, et al. High frequency of neurexin 1beta signal peptide structural variants in patients with autism. Neurosci. Lett. 2006;409:10–3.

62. Liu Y, Hu Z, Xun G, Peng Y, Lu L, Xu X, et al. Mutation analysis of the NRXN1 gene in a Chinese autism cohort. J Psychiatr Res. 2012;46:630–4.

63. Yan J, Noltner K, Feng J, Li W, Schroer R, Skinner C, et al. Neurexin 1alpha structural variants associated with autism. Neurosci. Lett. 2008;438:368–70.

64. Kim H-G, Kishikawa S, Higgins AW, Seong I-S, Donovan DJ, Shen Y, et al. Disruption of neurexin 1 associated with autism spectrum disorder. Am. J. Hum. Genet. 2008;82:199–207.

65. Camacho-Garcia RJ, Planelles MI, Margalef M, Pecero ML, Martínez-Leal R, Aguilera F, et al. Mutations affecting synaptic levels of neurexin-1β in autism and mental retardation. Neurobiol. Dis. 2012;47:135–43.

66. Koshimizu E, Miyatake S, Okamoto N, Nakashima M, Tsurusaki Y, Miyake N, et al. Performance comparison of bench-top next generation sequencers using microdroplet PCR-based enrichment for targeted sequencing in patients with autism spectrum disorder. PLoS ONE. 2013;8:e74167.

67. Autism Genome Project Consortium, Szatmari P, Paterson AD, Zwaigenbaum L, Roberts W, Brian J, et al. Mapping autism risk loci using genetic linkage and chromosomal rearrangements. Nat. Genet. 2007;39:319–28.

68. Prasad A, Merico D, Thiruvahindrapuram B, Wei J, Lionel AC, Sato D, et al. A discovery resource of rare copy number variations in individuals with autism spectrum disorder. G3 (Bethesda). 2012;2:1665–85.

69. Brandler WM, Antaki D, Gujral M, Noor A, Rosanio G, Chapman TR, et al. Frequency and Complexity of De Novo Structural Mutation in Autism. Am. J. Hum. Genet. 2016;98:667–79.

70. Tammimies K, Marshall CR, Walker S, Kaur G, Thiruvahindrapuram B, Lionel AC, et al. Molecular Diagnostic Yield of Chromosomal Microarray Analysis and Whole-Exome Sequencing in Children With Autism Spectrum Disorder. JAMA. 2015;314:895–903.

71. Zweier C, de Jong EK, Zweier M, Orrico A, Ousager LB, Collins AL, et al. CNTNAP2 and NRXN1 are mutated in autosomal-recessive Pitt-Hopkins-like mental retardation and determine the level of a common synaptic protein in Drosophila. Am. J. Hum. Genet. 2009;85:655–66.

72. Utine GE, Haliloğlu G, Volkan-Salancı B, Çetinkaya A, Kiper PÖ, Alanay Y, et al. Etiological yield of SNP microarrays in idiopathic intellectual disability. Eur. J. Paediatr. Neurol. 2014;18:327–37.

73. Duong L, Klitten LL, Møller RS, Ingason A, Jakobsen KD, Skjødt C, et al. Mutations in NRXN1 in a family multiply affected with brain disorders: NRXN1 mutations and brain disorders. Am. J. Med. Genet. B Neuropsychiatr. Genet. 2012;159B:354–8.

74. Imitola J, Walleigh D, Anderson CE, Jethva R, Carvalho KS, Legido A, et al. Fraternal twins with autism, severe cognitive deficit, and epilepsy: diagnostic role of chromosomal microarray analysis. Semin Pediatr Neurol. 2014;21:167–71.

75. Béna F, Bruno DL, Eriksson M, van Ravenswaaij-Arts C, Stark Z, Dijkhuizen T, et al. Molecular and clinical characterization of 25 individuals with exonic deletions of NRXN1 and comprehensive review of the literature. Am. J. Med. Genet. B Neuropsychiatr. Genet. 2013;162B:388–403.

76. Ching MSL, Shen Y, Tan W-H, Jeste SS, Morrow EM, Chen X, et al. Deletions of NRXN1 (neurexin-1) predispose to a wide spectrum of developmental disorders. Am. J. Med. Genet. B Neuropsychiatr. Genet. 2010;153B:937–47.

77. Lowther C, Speevak M, Armour CM, Goh ES, Graham GE, Li C, et al. Molecular characterization of NRXN1 deletions from 19,263 clinical microarray cases identifies exons important for neurodevelopmental disease expression. Genet. Med. 2016;

78. Piton A, Gauthier J, Hamdan FF, Lafrenière RG, Yang Y, Henrion E, et al. Systematic resequencing of X-chromosome synaptic genes in autism spectrum disorder and schizophrenia. Mol. Psychiatry. 2011;16:867–80.

79. Dibbens LM, Tarpey PS, Hynes K, Bayly MA, Scheffer IE, Smith R, et al. X-linked protocadherin 19 mutations cause female-limited epilepsy and cognitive impairment. Nat. Genet. 2008;40:776–81.

80. Marini C, Mei D, Parmeggiani L, Norci V, Calado E, Ferrari A, et al. Protocadherin 19 mutations in girls with infantile-onset epilepsy. Neurology. 2010;75:646–53.

81. Cappelletti S, Specchio N, Moavero R, Terracciano A, Trivisano M, Pontrelli G, et al. Cognitive development in females with PCDH19 gene-related epilepsy. Epilepsy Behav. 2015;42:36–40.

82. Higurashi N, Nakamura M, Sugai M, Ohfu M, Sakauchi M, Sugawara Y, et al. PCDH19-related female-limited epilepsy: further details regarding early clinical features and therapeutic efficacy. Epilepsy Res. 2013;106:191–9.

83. van Harssel JJT, Weckhuysen S, van Kempen MJA, Hardies K, Verbeek NE, de Kovel CGF, et al. Clinical and genetic aspects of PCDH19-related epilepsy syndromes and the possible role of PCDH19 mutations in males with autism spectrum disorders. Neurogenetics. 2013;14:23–34.

84. Marini C, Darra F, Specchio N, Mei D, Terracciano A, Parmeggiani L, et al. Focal seizures with affective symptoms are a major feature of PCDH19 gene-related epilepsy. Epilepsia. 2012;53:2111–9.

85. Dimova PS, Kirov A, Todorova A, Todorov T, Mitev V. A novel PCDH19 mutation inherited from an unaffected mother. Pediatr. Neurol. 2012;46:397–400.

86. Jamal SM, Basran RK, Newton S, Wang Z, Milunsky JM. Novel de novo PCDH19 mutations in three unrelated females with epilepsy female restricted mental retardation syndrome. Am. J. Med. Genet. A. 2010;152A:2475–81.

87. Hynes K, Tarpey P, Dibbens LM, Bayly MA, Berkovic SF, Smith R, et al. Epilepsy and mental retardation limited to females with PCDH19 mutations can present de novo or in single generation families. J. Med. Genet. 2010;47:211–6.

88. Kwong AK-Y, Fung C-W, Chan S-Y, Wong VC-N. Identification of SCN1A and PCDH19 mutations in Chinese children with Dravet syndrome. PLoS ONE. 2012;7:e41802.

89. Christian SL, Brune CW, Sudi J, Kumar RA, Liu S, Karamohamed S, et al. Novel submicroscopic chromosomal abnormalities detected in autism spectrum disorder. Biol. Psychiatry. 2008;63:1111–7.

90. Butler MG, Dasouki MJ, Zhou X-P, Talebizadeh Z, Brown M, Takahashi TN, et al. Subset of individuals with autism spectrum disorders and extreme macrocephaly associated with germline PTEN tumour suppressor gene mutations. J. Med. Genet. 2005;42:318–21.

91. Hobert JA, Embacher R, Mester JL, Frazier TW, Eng C. Biochemical screening and PTEN mutation analysis in individuals with autism spectrum disorders and macrocephaly. Eur. J. Hum. Genet. 2014;22:273–6.

92. Varga EA, Pastore M, Prior T, Herman GE, McBride KL. The prevalence of PTEN mutations in a clinical pediatric cohort with autism spectrum disorders, developmental delay, and macrocephaly. Genet. Med. 2009;11:111–7.

93. Goffin A, Hoefsloot LH, Bosgoed E, Swillen A, Fryns JP. PTEN mutation in a family with Cowden syndrome and autism. Am. J. Med. Genet. 2001;105:521–4.

94. McBride KL, Varga EA, Pastore MT, Prior TW, Manickam K, Atkin JF, et al. Confirmation study of PTEN mutations among individuals with autism or developmental delays/mental retardation and macrocephaly. Autism Res. 2010;3:137–41.

95. Vanderver A, Tonduti D, Kahn I, Schmidt J, Medne L, Vento J, et al. Characteristic brain magnetic resonance imaging pattern in patients with macrocephaly and PTEN mutations. Am. J. Med. Genet. A. 2014;164A:627–33.

96. Frazier TW, Embacher R, Tilot AK, Koenig K, Mester J, Eng C. Molecular and phenotypic abnormalities in individuals with germline heterozygous PTEN mutations and autism. Mol. Psychiatry. 2015;20:1132–8.

97. Weiss LA, Escayg A, Kearney JA, Trudeau M, MacDonald BT, Mori M, et al. Sodium channels SCN1A, SCN2A and SCN3A in familial autism. Mol. Psychiatry. 2003;8:186–94.

98. O’Roak BJ, Vives L, Girirajan S, Karakoc E, Krumm N, Coe BP, et al. Sporadic autism exomes reveal a highly interconnected protein network of de novo mutations. Nature. 2012;485:246–50.

99. Tan EH, Yusoff AAM, Abdullah JM, Razak SA. Generalized epilepsy with febrile seizure plus (GEFS+) spectrum: Novel de novo mutation of SCN1A detected in a Malaysian patient. J Pediatr Neurosci. 2012;7:123–5.

100. Frosk P, Mhanni AA, Rafay MF. SCN1A mutation associated with intractable myoclonic epilepsy and migraine headache. J. Child Neurol. 2013;28:389–91.

101. Osaka H, Ogiwara I, Mazaki E, Okamura N, Yamashita S, Iai M, et al. Patients with a sodium channel alpha 1 gene mutation show wide phenotypic variation. Epilepsy Res. 2007;75:46–51.

102. Veeramah KR, O’Brien JE, Meisler MH, Cheng X, Dib-Hajj SD, Waxman SG, et al. De novo pathogenic SCN8A mutation identified by whole-genome sequencing of a family quartet affected by infantile epileptic encephalopathy and SUDEP. Am. J. Hum. Genet. 2012;90:502–10.

103. Blanchard MG, Willemsen MH, Walker JB, Dib-Hajj SD, Waxman SG, Jongmans MCJ, et al. De novo gain-of-function and loss-of-function mutations of SCN8A in patients with intellectual disabilities and epilepsy. J. Med. Genet. 2015;52:330–7.

104. Yuen RKC, Thiruvahindrapuram B, Merico D, Walker S, Tammimies K, Hoang N, et al. Whole-genome sequencing of quartet families with autism spectrum disorder. Nat. Med. 2015;21:185–91.

105. Sanders SJ, Murtha MT, Gupta AR, Murdoch JD, Raubeson MJ, Willsey AJ, et al. De novo mutations revealed by whole-exome sequencing are strongly associated with autism. Nature. 2012;485:237–41.

106. Deciphering Developmental Disorders Study. Large-scale discovery of novel genetic causes of developmental disorders. Nature. 2015;519:223–8.

107. Rauch A, Wieczorek D, Graf E, Wieland T, Endele S, Schwarzmayr T, et al. Range of genetic mutations associated with severe non-syndromic sporadic intellectual disability: an exome sequencing study. Lancet. 2012;380:1674–82.

108. Tavassoli T, Kolevzon A, Wang AT, Curchack-Lichtin J, Halpern D, Schwartz L, et al. De novo SCN2A splice site mutation in a boy with Autism spectrum disorder. BMC Med. Genet. 2014;15:35.

109. Horvath GA, Demos M, Shyr C, Matthews A, Zhang L, Race S, et al. Secondary neurotransmitter deficiencies in epilepsy caused by voltage-gated sodium channelopathies: A potential treatment target? Mol. Genet. Metab. 2016;117:42–8.

110. Kamiya K, Kaneda M, Sugawara T, Mazaki E, Okamura N, Montal M, et al. A nonsense mutation of the sodium channel gene SCN2A in a patient with intractable epilepsy and mental decline. J. Neurosci. 2004;24:2690–8.

111. Coe BP, Witherspoon K, Rosenfeld JA, van Bon BWM, Vulto-van Silfhout AT, Bosco P, et al. Refining analyses of copy number variation identifies specific genes associated with developmental delay. Nat. Genet. 2014;46:1063–71.

112. Hansen J, Snow C, Tuttle E, Ghoneim DH, Yang C-S, Spencer A, et al. De novo mutations in SIK1 cause a spectrum of developmental epilepsies. Am. J. Hum. Genet. 2015;96:682–90.

113. Merner ND, Chandler MR, Bourassa C, Liang B, Khanna AR, Dion P, et al. Regulatory domain or CpG site variation in SLC12A5, encoding the chloride transporter KCC2, in human autism and schizophrenia. Front Cell Neurosci. 2015;9:386.

114. Lopes F, Barbosa M, Ameur A, Soares G, de Sá J, Dias AI, et al. Identification of novel genetic causes of Rett syndrome-like phenotypes. J. Med. Genet. 2016;

115. Carvill GL, McMahon JM, Schneider A, Zemel M, Myers CT, Saykally J, et al. Mutations in the GABA Transporter SLC6A1 Cause Epilepsy with Myoclonic-Atonic Seizures. Am. J. Hum. Genet. 2015;96:808–15.

116. Romaniello R, Saettini F, Panzeri E, Arrigoni F, Bassi MT, Borgatti R. A de-novo STXBP1 gene mutation in a patient showing the Rett syndrome phenotype. Neuroreport. 2015;26:254–7.

117. Stamberger H, Nikanorova M, Willemsen MH, Accorsi P, Angriman M, Baier H, et al. STXBP1 encephalopathy: A neurodevelopmental disorder including epilepsy. Neurology. 2016;86:954–62.

118. Campbell IM, Yatsenko SA, Hixson P, Reimschisel T, Thomas M, Wilson W, et al. Novel 9q34.11 gene deletions encompassing combinations of four Mendelian disease genes: STXBP1, SPTAN1, ENG, and TOR1A. Genet. Med. 2012;14:868–76.

119. Redin C, Gérard B, Lauer J, Herenger Y, Muller J, Quartier A, et al. Efficient strategy for the molecular diagnosis of intellectual disability using targeted high-throughput sequencing. J. Med. Genet. 2014;51:724–36.

120. Van Balkom IDC, Vuijk PJ, Franssens M, Hoek HW, Hennekam RCM. Development, cognition, and behaviour in Pitt-Hopkins syndrome. Dev Med Child Neurol. 2012;54:925–31.
