# Supplementary material for: Autism spectrum disorder and epileptic encephalopathy: common causes, many questions
Source: J Neurodev Disord. 2017 Jun 23;9:23. doi: 10.1186/s11689-017-9202-0 (PMC5481888; doi:10.1186/s11689-017-9202-0)
Supplement: Supplementary file 3 — Functions of epileptic encephalopathy genes linked to ASD. (DOCX 19 kb) [file 11689_2017_9202_MOESM3_ESM.docx]

## Table S3. Functions of epileptic encephalopathy genes linked to ASD.

| **Gene Symbol** | **Gene Full Name** | **Gene Functions** |
| --- | --- | --- |
| *ARX* | aristaless related homeobox | -encodes a transcription factor  -plays a role in CNS development |
| *CACNA1A* | calcium voltage-gated channel subunit alpha1 A | -encodes a subunit of a voltage-gated Ca^++^ channel  -facilitates Ca^++^ entry into cells |
| *CDKL5* | cyclin dependent kinase like 5 | -encodes a protein kinase  -helps regulate the activity of genes implicated in brain development |
| *CHD2* | chromodomain helicase DNA binding protein 2 | -encodes a DNA-binding protein  -helps modify chromatin structure, regulating transcription |
| *ERBB4* | erb-b2 receptor tyrosine kinase 4 | -encodes a tyrosine kinase  -helps induce mitogenesis and cellular differentiation |
| *FLNA* | filamin A | -encodes an actin-binding protein  -helps remodel cytoskeletal structure |
| *FOXG1* | forkhead box G1 | -encodes a transcription factor  -helps regulate brain development |
| *GABRA1* | gamma-aminobutyric acid type A receptor alpha1 subunit | -encodes a subunit of a GABA_A_ receptor  -plays a role in GABAergic neurotransmission |
| *GABRB1* | gamma-aminobutyric acid type A receptor beta1 subunit | -encodes a subunit of a GABA_A_ receptor  -plays a role in GABAergic neurotransmission |
| *GABRB3* | gamma-aminobutyric acid type A receptor beta3 subunit | -encodes a subunit of a GABA_A_ receptor  -plays a role in GABAergic neurotransmission |
| *GABRG2* | gamma-aminobutyric acid type A receptor gamma2 subunit | -encodes a subunit of a GABA_A_ receptor  -plays a role in GABAergic neurotransmission |
| *GRIN1* | glutamate ionotropic receptor NMDA type subunit 1 | -encodes subunit of an N-methyl-D-aspartate receptor  -plays a role in synaptic plasticity |
| *GRIN2A* | glutamate ionotropic receptor NMDA type subunit 2A | -encodes subunit of an N-methyl-D-aspartate receptor  -plays a role in synaptic plasticity |
| *GRIN2B* | glutamate ionotropic receptor NMDA type subunit 2B | -encodes a subunit of an N-methyl-D-aspartate receptor  -plays a role in synaptic plasticity |
| *HCN1* | hyperpolarization activated cyclic nucleotide gated potassium channel 1 | -encodes a hyperpolarization-activated cation channel  -helps create rhythmic currents in nerve and heart cells |
| *IQSEC2* | IQ motif and Sec7 domain 2 | -encodes a guanine nucleotide exchange factor  -helps organize the cytoskeleton and synapse |
| *KCNQ2* | potassium voltage-gated channel subfamily Q member 2 | -encodes a subunit of a voltage-gated K^+^ channel  -helps determine neuronal excitability |
| *KCNQ3* | potassium voltage-gated channel subfamily Q member 3 | *-*encodes a subunit of a voltage-gated K^+^ channel  -helps determine neuronal excitability |
| *MEF2C* | myocyte enhancer factor 2C | -encodes a transcription enhancer  -plays a role in myogenesis |
| *MTOR* | mechanistic target of rapamycin | -encodes a phosphatidylinositol kinase-related kinase  -serves as a target of the FKBP12-rapamycin complex mediating the cell cycle |
| *NRXN1* | neurexin 1 | -encodes a cell-surface receptor  -binds neuroligins at synapses, facilitating neurotransmission |
| *PCDH19* | protocadherin 19 | -encodes a protocadherin  -mediates cell-adhesion, mainly in the brain |
| *PLCB1* | phospholipase C beta 1 | -encodes an enzyme that catalyzes the conversion of phosphatidylinositol 4,5-bisphosphate into  inositol 1,4,5-trisphosphate and diacylglycerol  -facilitates signal transduction |
| *PTEN* | phosphatase and tensin homolog | -encodes a phosphatase  -helps regulate signal transduction pathways |
| *SCN1A* | sodium voltage-gated channel alpha subunit 1 | -encodes a subunit of a voltage-gated Na^+^ channel  -helps generate and propagate action potentials |
| *SCN8A* | sodium voltage-gated channel alpha subunit 8 | -encodes the pore of a voltage-gated Na^+^ channel  -plays a role in rapid membrane depolarization |
| *SCN2A* | sodium voltage-gated channel alpha subunit 2 | -encodes a subunit of a voltage-gated Na^+^ channel  -plays a role in potential generation and propagation |
| *SETBP1* | SET binding protein 1 | -encodes a SET oncogene binding protein  -plays a role in DNA replication |
| *SIK1* | salt inducible kinase 1 | -encodes an adenosine monophosphate kinase  -facilitates signal transduction |
| *SLC12A5* | solute carrier family 12 member 5 | -encodes an integral membrane K^+^-Cl^-^ cotransporter  -helps regulate neuronal chloride concentration |
| *SLC35A2* | solute carrier family 35 member A2 | -encodes a nucleotide-sugar transporter  -plays a role in the glycosylation pathway |
| *SLC6A1* | solute carrier family 6 member 1 | -encodes a GABA transporter  -plays a role in synaptic GABA re-uptake |
| *STXBP1* | syntaxin binding protein 1 | -encodes a syntaxin-binding protein  -helps regulate neurotransmitter release |
| *TCF4* | transcription factor 4 | -encodes a transcription factor  -plays a role in CNS development |

For each gene, full name and function description is based on corresponding entries in Entrez Gene (<http://www.ncbi.nlm.nih.gov/gene>) and/or Online Mendelian Inheritance in Man ([http://www.omim.org](http://www.omim.org/)).

## Abbreviations

- ASD = autism spectrum disorder
- CNS = central nervous system
- GABA = γ-aminobutyric acid
